# Engineered RGD‐Treg‐Exos Targeted Delivery of miR‐218‐5p to Activate Mitophagy and Attenuate Podocyte Injury in Diabetic Kidney Disease

**DOI:** 10.1002/advs.202412034

**Published:** 2025-08-19

**Authors:** Zhaochen Guo, Shaohui Gao, Ziyue Wang, Zige Chen, Jinglei Chen, Aiping Duan, Feng Xu, Qinger Wang, Weisong Qin, Caihong Zeng, Zhihong Liu, Hao Bao

**Affiliations:** ^1^ National Clinical Research Center for Kidney Diseases Jinling Hospital Affiliated Hospital of Medical School Nanjing University Nanjing 210016 China; ^2^ State Key Laboratory of Pharmaceutical Biotechnology Medical School Nanjing University Nanjing 210093 China; ^3^ Department of Urology Jinling Hospital, Affiliated Hospital of Medical School Nanjing University Nanjing 210016 China

**Keywords:** diabetic kidney disease, exosome, miRNA, mitophagy, treg

## Abstract

Diabetic kidney disease (DKD) is the main cause of end‐stage kidney disease, and podocyte injury is an important factor in the development of DKD. Mitophagy is severely inhibited in the podocytes of patients. Damaged mitochondria aggregate in the cytoplasm and can not be removed effectively. Restoring mitophagy may be a novel strategy for the treatment of DKD. In this study, Regulatory T cells (Tregs) are found to reduce podocyte injury in DKD through exosomes. Sequencing and cross‐sectional analysis revealed that exosomes from Tregs delivered miR‐218‐5p to increase mitophagy in podocytes by inhibiting the TNC/TLR4/SRC/FUNDC1 pathway. Treg‐Exos are engineered to express RGD peptides on the membrane surface. RGD‐Treg‐Exos bind to integrins on the surface of podocytes and effectively target podocytes for the delivery of miR‐218‐5p, thus increasing mitophagy in podocytes, reducing cell apoptosis, and alleviating podocyte injury. In summary, this study revealed that engineered RGD‐Treg‐Exos effectively ameliorated podocyte injury in DKD, thus constituting a novel method for DKD treatment.

## Graphical abstract

Tregs‐Exos deliver miR‐218‐5p to increase mitophagy in podocytes by inhibiting the TNC/TLR4/SRC/FUNDC1 pathway. RGD‐Treg‐Exos, engineered to express RGD peptides on the membrane surface, bind to integrins on the surface of podocytes and effectively target podocytes for the delivery of miR‐218‐5p, which increases mitophagy and reduces apoptosis in podocytes. In all, Engineered RGD‐Treg‐Exos effectively alleviate podocyte injury and represent a novel method for the treatment of DKD.

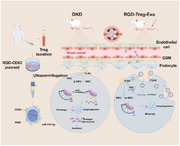

## 1 Introduction

Diabetic kidney disease (DKD) is one of the most common and serious complications of diabetes. The main feature of DKD is glomerular filtration barrier (GFB) dysfunction, which leads to the leakage of proteins, metabolites, and ions into the urine and eventual development of end‐stage kidney disease (ESKD).^[^ ^1^, ^2^ ^]^ Podocytes are located outside the glomerular basement membrane (GBM), ensuring the mechanical stability of the GFB and preventing protein loss into the urine. Podocyte dysfunction is one of the earliest glomerular morphological changes and plays a key role in the progression of DKD.^[^ ^3^ ^]^ High glucose (HG) and advanced glycation end product (AGEs) levels in the body can lead to glomerular podocyte injury and even podocyte detachment from the GBM through various pathways, such as the inhibition of mitophagy, promotion of lipid aggregation, generation of reactive oxygen species, and endoplasmic reticulum stress, resulting in reduced podocyte density and weakened GFB function, which leads to proteinuria and further promotes the development of DKD.^[^ ^4^ ^]^ Although podocyte injury is a key link in DKD, treatment options are currently limited.

Mitophagy is a type of selective autophagy that clears damaged mitochondria. Mitochondrial phagosomes fuse with lysosomes to degrade the mitochondrial phagosome content. Autophagy contributes to cellular homeostasis and protein quality control.^[^ ^5^ ^]^ In recent years, studies have indicated that the basal mitophagy rate in the kidney is greater than that in other organs and that it plays a key role in maintaining body homeostasis.^[^ ^6^, ^7^ ^]^ In addition, podocytes are rich in mitochondria and rely heavily on them to provide energy to maintain normal function. Dysregulation of mitophagy in podocytes can cause or aggravate DKD.

Regulatory T cells (Tregs) are a group of T cell subsets that express Foxp3, CD25, and CD4 as their cell phenotypes, have negative immunoregulatory functions, and can suppress the immune response of other immune cells. Tregs can regulate the biological functions of target cells through direct contact and the secretion of exosomes or cytokines.^[^ ^8^ ^]^ As a novel cell‐free therapy, Treg‐Exos do not carry the risk of Treg phenotype switching and can easily cross barrier structures such as the blood‐brain barrier and glomerular filtration barrier. Through their unique immune regulatory function, Tregs and Treg‐Exos not only play a key role in protecting the body from autoimmune attack and inhibiting immune escape but also improve the repair and regeneration of damaged tissue.^[^ ^9^, ^10^, ^11^ ^]^ So far, there have been few studies on Tregs and Treg‐Exos that protect against kidney damage. They have been shown to prevent the development of autoimmune kidney diseases in various models, including glomerulonephritis in lupus‐prone mice,^[^ ^12^ ^]^ pristane‐induced lupus nephritis,^[^ ^13^ ^]^ anti‐GBM antibody‐induced glomerulonephritis,^[^ ^14^ ^]^ and IgA nephropathy.^[^ ^15^ ^]^ In addition, the protective effects of Treg cells have also been shown in rodent models of acute kidney injury (AKI), such as ischemia‐reperfusion injury,^[^ ^16^ ^]^ cisplatin nephrotoxicity,^[^ ^17^ ^]^ and sepsis‐induced AKI,^[^ ^18^ ^]^ and in models of chronic kidney disease, including adriamycin nephropathy.^[^ ^19^ ^]^ Furthermore, a significant reduction in infiltrating Treg cells in diabetic kidney tissues has been confirmed by multiple fluorescence staining.^[^ ^20^ ^]^ In cardiorenal syndrome, myocardial infarction accelerates glomerular injury and microalbuminuria in diabetic rats by altering the Treg/Th17 ratio.^[^ ^21^ ^]^ Adoptive transfer of Treg cells prevents the progression of diabetic kidney disease in a db/db mouse model.^[^ ^22^ ^]^ However, the therapeutic effect and mechanism of Tregs in podocyte injury in DKD remain unclear, and further exploration is urgently needed.

## 2 Results

### 2.1 Tregs and Treg‐Exos Reduce Podocyte Injury

**Figure 1:**
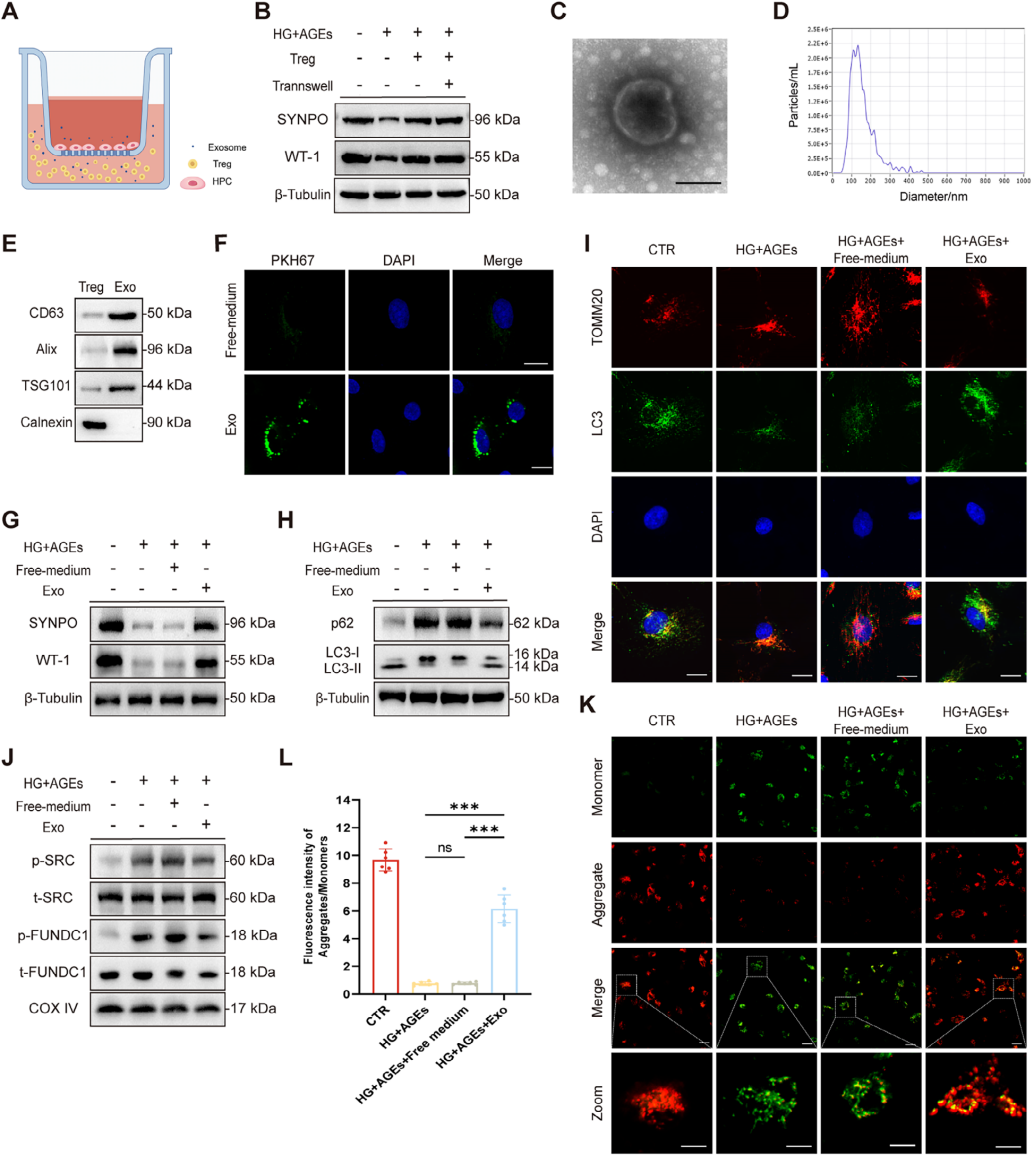
Tregs and Treg‐Exos alleviate podocyte injury induced by HG+AGEs. A) Schematic diagram of the co‐culture of Tregs and HPCs. B) Western blot analysis of SYNPO and WT‐1 expression in HPCs after co‐culture with Tregs for 48 h. *n* = 3 per group. C) Identification of Treg‐Exos by TEM and D) NTA. Scale bar, 100 nm. *n* = 3 per group. E) Western blot analysis of Treg‐Exos biomarkers (CD63, Alix, and TSG101) and the negative control (calnexin). *n* = 3 per group. F) Fluorescent staining images of PKH67‐labeled Treg‐Exos ingested by HPCs after 48 h of incubation. *n* = 3 per group. G) Western blot analysis of SYNPO, WT‐1, p62 expression, and LC3 conversion, H) in HPCs treated with free medium or Treg‐Exos for 48 h. *n* = 3 per group. I) Immunofluorescence staining images of TOMM20 and LC3 expression in HPCs treated with free medium or Treg‐Exos for 48 h. Scale bar, 20 nm. *n* = 3 per group. J) Western blot analysis of p‐SRC, t‐SRC, p‐FUNDC1, and t‐FUNDC1 expression in HPCs treated with free medium or Treg‐Exos for 48 h. *n* = 3 per group. K) Fluorescence images of mitochondrial membrane potential by JC‐1 staining in HPCs treated with free medium or Treg‐Exos for 48 h. Scale bar, 20 nm (Merge), 2 nm (Zoom). *n* = 6 per group. L) Quantitative statistics of the fluorescence intensity of aggregates/monomers in (K) results. *n* = 6 per group. Images represent one representative experiment from three to six independent experiments. For all statistical plots, the data are presented as mean ± SD, and the distinct dots are represented as the individual values of 6 replicates. *p* values were calculated by one‐way ANOVA and Tukey's multiple comparison test. ns *p >0.05*, ^***^p < 0.001.

We used HG+AGEs to model the diabetic microenvironment,^[^ ^23^, ^24^ ^]^ which resulted in decreased expression of SYNPO and WT1 and increased cell apoptosis. To explore the effects of Tregs on human podocytes, we isolated Tregs from the peripheral blood of healthy individuals and cultured them in vitro (Figure S1A, Supporting Information). Co‐culture experiments revealed that, regardless of the Transwell system used (**Figure** **1**A), Tregs effectively restored the expression of SYNPO and WT1 in podocytes (Figure 1B) and reduced apoptosis (Figure S1B, Supporting Information). Treg‐Exos were obtained via ultracentrifugation. Transmission electron microscopy (TEM) revealed that the exosomes had a disc‐shaped double‐layer membrane structure (Figure 1C). Nanoparticle tracking analysis (NTA) revealed that the peak diameter of the exosomes was 130.3 nm, which was consistent with the size of the exosomes (30–150 nm) (Figure 1D). Western blot (WB) analysis confirmed that Treg‐Exos expressed high levels of CD63, Alix, and TSG 101, and low levels of calnexin (Figure 1E).

Treg‐Exos were labelled with PKH67 and co‐cultured with human podocytes. Efficient uptake of Treg‐Exos by human podocytes was observed by immunofluorescence (IF) (Figure 1F). WB results revealed that Treg‐Exos significantly reversed the decreased expression of SYNPO and WT1 caused by HG+AGEs, whereas Treg‐free medium had no effect on SYNPO or WT‐1 expression (Figure 1G). Treg‐Exos reduced the level of the autophagy substrate SQSTM1/p62 in podocytes, improved the conversion of intracellular LC3I to LC3II, increased the number of autophagosomes, reduced the number of damaged mitochondria, and restored mitophagy (Figure 1H,I). Moreover, WB analysis of mitophagy signalling pathways, including the PINK/Parkin, BNIP3, Nix, and FUNDC1 pathways (Figure S1C, Supporting Information), revealed that Treg‐Exos significantly inhibited the SRC/FUNDC1 pathway (Figure 1J). JC‐1 staining showed that Treg‐Exos restored mitochondrial membrane potential (Figure 1K,L). Additionally, Treg‐Exos, but not Treg‐free medium, significantly inhibited podocyte apoptosis (Figure S1D, Supporting Information) induced by HG + AGEs stimulation. In the Treg and podocyte co‐culture system, the protective effect of Tregs on podocytes disappeared after inhibition of exosome secretion with GW4689 (Figure S1E, Supporting Information).

**Figure 2:**
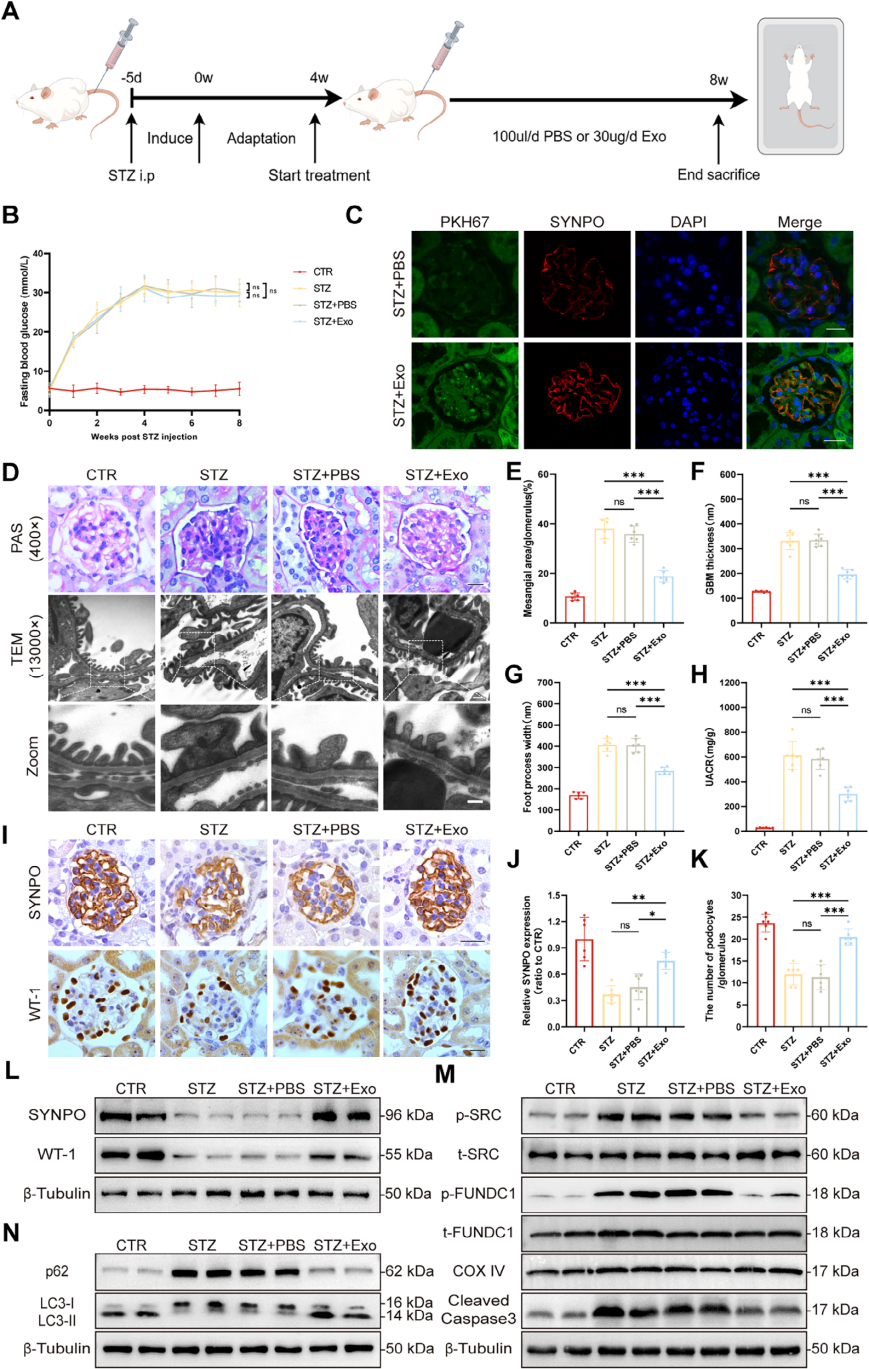
Treg‐Exos alleviated podocyte injury in diabetic mice in vivo. A) Schematic diagram of the evaluation of podocyte injury in Treg‐Exos injected diabetic mice. B) Results of the weekly fasting blood glucose test in diabetic mice injected with Treg‐Exos. *n* = 6 per group. C) Colocalization fluorescence staining images of PKH67‐labeled Treg‐Exos (green) and the podocyte marker SYNPO (red). Scale bar, 20 µm. *n* = 3 per group. D) Morphological examination of glomerular changes using PAS staining and TEM in diabetic mice injected with Treg‐Exos. Scale bar, 20 µm (PAS), 250 nm (TEM). *n* = 6 per group. E) Quantification of the mesangial area in diabetic mice injected with Treg‐Exos. *n* = 6 per group. F) Quantification of GBM thickness in diabetic mice injected with Treg‐Exos. *n* = 6 per group. G) Mean width of the podocyte foot processes in diabetic mice injected with Treg‐Exos. *n* = 6 per group. H) Urinary albumin‐to‐creatinine ratios in diabetic mice injected with Treg‐Exos. *n* = 6 per group. I) IHC analysis of SYNPO and WT‐1 expression in glomeruli of diabetic mice injected with Treg‐Exos. Scale bar, 20 µm. *n* = 6 per group. J) Quantitative analysis of SYNPO density in (I). *n* = 6 per group. K) Quantitative analysis of podocyte numbers per glomerulus in (I). *n* = 6 per group. Western blot analysis of SYNPO, WT‐1 L), p‐SRC, t‐SRC, p‐FUNDC1, t‐FUNDC1, cleaved caspase3 M), p62 expression, and LC3 conversion N) in renal cortex of diabetic mice injected with Treg‐Exos. *n* = 3 per group. Images represent one representative experiment from three to six independent experiments. For all statistical plots, the data are presented as mean ± SD, and the distinct dots are represented as the individual values of 6 replicates. *p* values were calculated by one‐way ANOVA and Tukey's multiple comparison test. ns *p >0.05*, ^*^p < 0.05, ^**^p < 0.01, ^***^p < 0.001.

We generated a DKD mouse model via a high‐fat diet (HFD) and intraperitoneal injection of streptozotocin (STZ), and then treated the mice with Treg‐Exos (**Figure** **2**A).

Blood glucose monitoring showed no significant changes in the mouse blood glucose levels (Figure 2B). Cryosections of kidney tissues revealed that Treg‐Exos could be taken up by podocytes (Figure 2C). Periodic acid‐Schiff (PAS) and TEM revealed that mesangial widening, basement membrane thickening, and foot process fusion were reversed in the Treg‐Exos group (Figure 2D–G). Moreover, Treg‐Exos significantly reduced the urine albumin‐creatinine ratio (UACR) in diabetic mice (Figure 2H). Additionally, immunohistochemistry (IHC) and WB results revealed that the expression of SYNPO and WT1 in the Treg‐Exos group was increased (Figure 2I–L), and that the level of mitophagy was restored (Figure 2M,N; Figure S2A,B, Supporting Information). Besides, the activation of caspase‐3 was significantly reduced (Figure 2M).

### 2.2 Screening of Candidate MicroRNAs (miRNAs)

**Figure 3:**
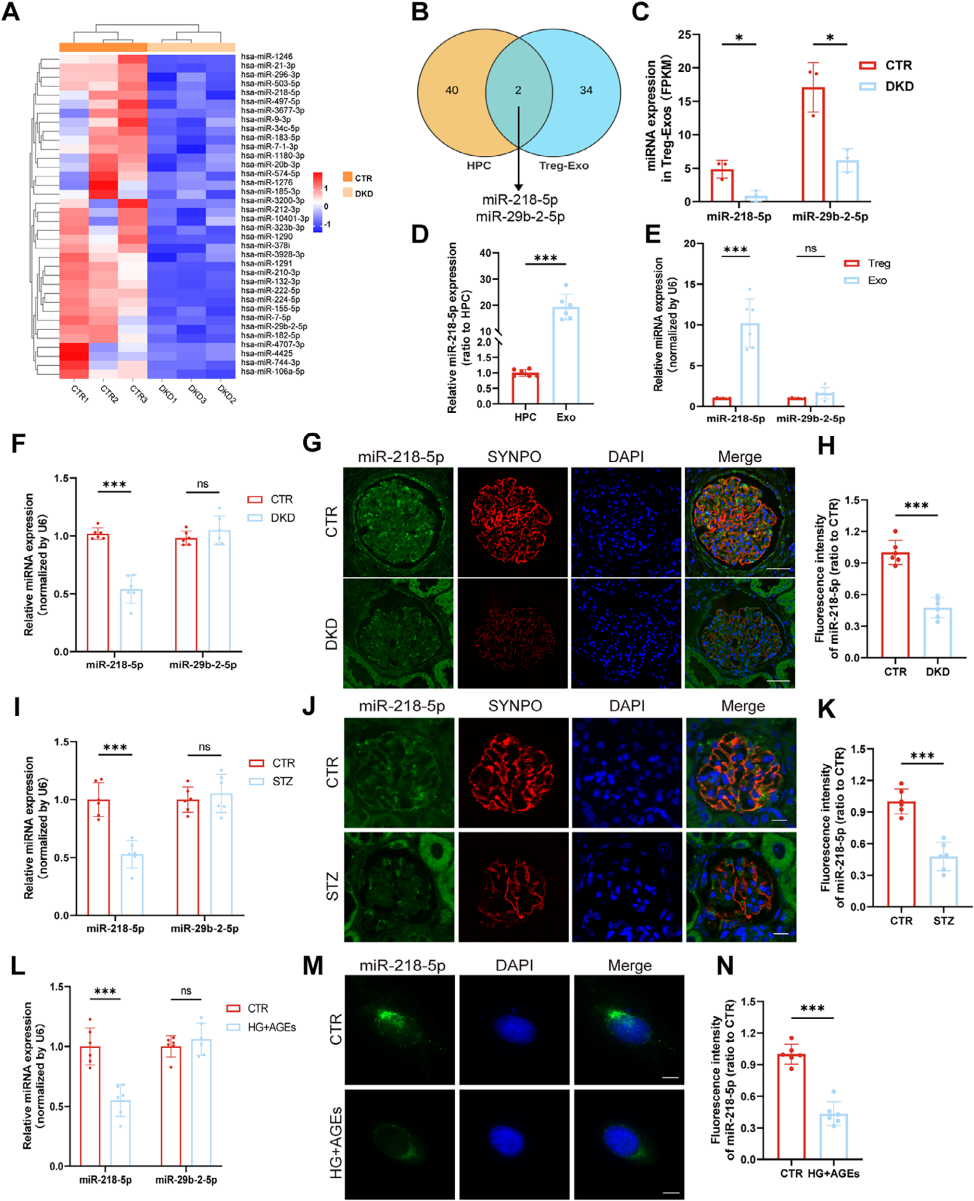
Treg‐Exos alleviates podocyte injury via miR‐218‐5p. A) Heatmap of upregulated miRNAs in Treg‐Exos of healthy volunteers compared to that of DKD patients. B) Venn diagram illustrating the intersection of upregulated miRNAs in Treg‐Exos of healthy volunteers and downregulated miRNAs in HPCs treated with HG+AGEs. C) Quantitative analysis of miR‐218‐5p and miR‐29b‐2‐5p expression in Treg‐Exos by FPKM. n = 3 per group. D) Quantitative PCR analysis of the expression of miR‐218‐5p in HPCs and Treg‐Exos. *n* = 6 per group. E) Quantitative PCR analysis of miR‐218‐5p and miR‐29b‐2‐5p expression in Tregs and Treg‐Exos. *n* = 6 per group. Quantitative PCR analysis of the expression of miR‐218‐5p and miR‐29b‐2‐5p in DKD patients (F), diabetic mice (I), and HPCs treated with HG+AGEs (L). *n* = 6 per group. Fluorescence in situ hybridization images of miR‐218‐5p in DKD patients (G), diabetic mice (J), and HPCs treated with HG+AGEs (M). *n* = 6 per group. (H, K, N) Quantitative statistics of fluorescence intensity of the miR‐218‐5p probe in (G, J, M) results. *n* = 6 per group. Images represent one representative experiment from six independent experiments. For all statistical plots, the data are presented as mean ± SD, and the distinct dots are represented as the individual values of 6 replicates. *p* values were calculated by a two‐tailed unpaired Student's *t* test. ns *p >0.05*, ^*^p < 0.05, ^***^p < 0.001.

To screen for miRNAs involved in podocyte injury in DKD, we performed miRNA sequencing on human podocytes and Treg‐Exos (**Figure** **3**A; Figure S3A, Supporting Information). The intersection of miRNAs downregulated in the HG+AGEs group and miRNAs enriched in Treg‐Exos from healthy controls was obtained. We found that two miRNAs, miR‐218‐5p and miR‐29b‐2‐5p, may be involved in the mechanism underlying the efficacy of Treg‐Exos (Figure 3B). Figure 3C shows the FPKM of miRNAs in Treg‐Exos from healthy controls and patients with DKD. Moreover, the expression level of miR‐218‐5p in Treg‐Exos was much higher than that in human podocytes (Figure 3D). Real‐time quantitative polymerase chain reaction (RT‐qPCR) analysis of Tregs and Treg‐Exos revealed that miR‐218‐5p was highly enriched in Treg‐Exos, whereas there was low enrichment of miR‐29b‐2‐5p (Figure 3E). RT‐qPCR and in situ hybridization showed that the level of miR‐218‐5p was decreased in kidney biopsy samples from DKD patients (Figure 3F–H), renal tissues of diabetic mice (Figure 3I–K), and HG+AGEs treated human podocytes (Figure 3L–N). Compared to treatment with HG or AGEs alone, the level of miR‐218‐5p was significantly reduced in human podocytes treated with HG+AGEs (Figure S3B, Supporting Information). In addition, we found that the level of miR‐218‐5p in Treg‐Exos from healthy controls was downregulated after treatment with HG+AGEs (Figure S3C, Supporting Information), while that in DKD patients returned to normal after treatment with NG (Figure S3D, Supporting Information).

We speculated that miR‐218‐5p plays an important role in the Treg‐Exos treatment of podocyte injury.

### 2.3 Treg‐Exos^miR‐inhibition^ Abolish the Protective Effect of Treg‐Exos on Podocytes

**Figure 4:**
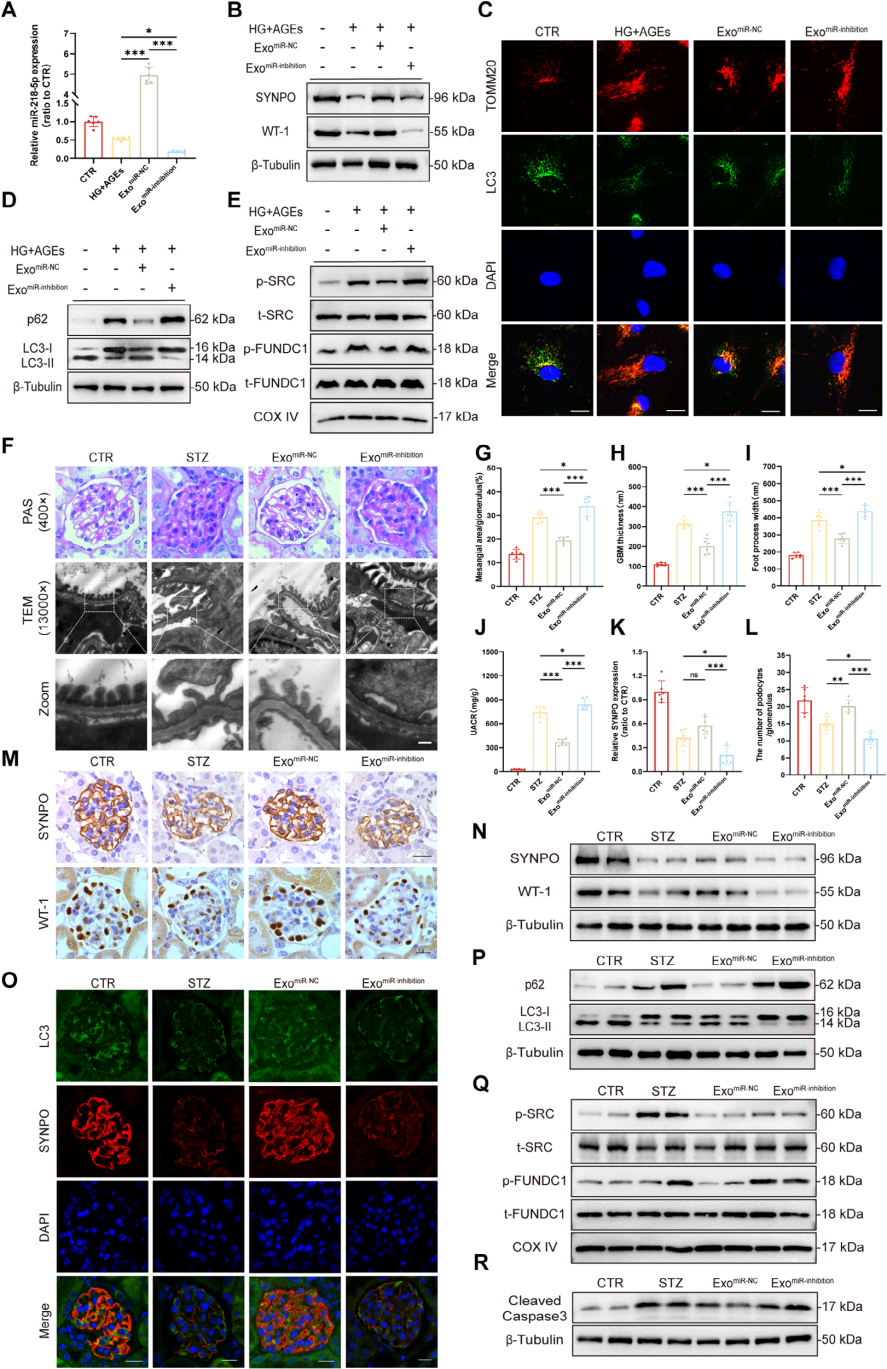
Treg‐Exos^miR‐inhibition^ abolish the protective effect of Treg‐Exos on podocytes. A) Quantitative PCR analysis of the expression of miR‐218‐5p in HPCs treated with Treg‐Exos^miR‐inhibition^ for 24 h. n = 6 per group. B) Western blot analysis of SYNPO and WT‐1 expression in HPCs treated with Treg‐Exos^miR‐inhibition^ for 48 h. *n* = 3 per group. C) Immunofluorescence staining images of TOMM20 and LC3 expression in HPCs treated with Treg‐Exos^miR‐inhibition^ for 48 h. Scale bar,20 nm. n = 3 per group. Western blot analysis of p62 expression, LC3 conversion (D), p‐SRC, t‐SRC, p‐FUNDC1, and t‐FUNDC1 expression (E) in HPCs treated with Treg‐Exos^miR‐inhibition^ for 48 h. *n* = 3 per group. F) Morphological examination of glomerular changes using PAS staining and TEM in diabetic mice injected with Treg‐Exos^miR‐inhibition^. Scale bar, 20 µm (PAS), 250 nm (TEM), 25 nm (Zoom). n = 6 per group. G) Quantification of the mesangial area in diabetic mice injected with Treg‐Exos^miR‐inhibition^. *n* = 6 per group. H) Quantification of GBM thickness in diabetic mice injected with Treg‐Exos^miR‐inhibition^. *n* = 6 per group. I) Mean width of the podocyte foot processes in diabetic mice injected with Treg‐Exos^miR‐inhibition^. *n* = 6 per group. J) Urinary albumin‐to‐creatinine ratios in diabetic mice injected with Treg‐Exos^miR‐inhibition^. *n* = 6 per group. M) IHC analysis of SYNPO and WT‐1 expression in the glomerular of diabetic mice injected with Treg‐Exos^miR‐inhibition^. Scale bar, 20 µm. *n* = 6 per group. K) Quantitative analysis of SYNPO density in (M). *n* = 6 per group. L) Quantitative analysis of the number of podocytes per glomerulus (M). *n* = 6 per group. N) Western blot analysis of SYNPO and WT‐1 expression in the renal cortex of diabetic mice injected with Treg‐Exos^miR‐inhibition^. *n* = 3 per group. O) Immunofluorescence staining images of LC3 and SYNPO expression in the glomerular of diabetic mice injected with Treg‐Exos^miR‐inhibition^. Scale bar, 20 µm. *n* = 3 per group. Western blot analysis of p62 expression, LC3 conversion (P), p‐SRC, t‐SRC, p‐FUNDC1, t‐FUNDC1 (Q), and cleaved caspase3 expression (R) in renal cortex of diabetic mice injected with Treg‐Exos^miR‐inhibition^. *n* = 3 per group. Images represent one representative experiment from three to six independent experiments. For all statistical plots, the data are presented as mean ± SD, and the distinct dots are represented as the individual values of 6 replicates. *p* values were calculated by one‐way ANOVA and Tukey's multiple comparison test. ns *p >0.05*, ^*^p < 0.05, ^**^p < 0.01, ^***^p < 0.001.

To investigate the protective effect of miR‐218‐5p‐mediated Treg‐Exos on podocytes in DKD, we knocked down miR‐218‐5p in Treg‐Exos using the miR‐218‐5p‐inhibition plasmids (Figure S4A, Supporting Information). In vitro experiments showed that Treg‐Exos^miR‐218‐5p‐inhibition^ (hereinafter referred to as Treg‐Exos^miR‐inhibition^ or Exos^miR‐inhibition^) abolished the protection of podocytes by Treg‐Exos, including lower SYNPO and WT1 expression (**Figure** **4**A,B), inhibited mitophagy (Figure 4C–E), and increased apoptosis (Figure S4B, Supporting Information). Similarly, in vivo experiments, PAS and TEM analyses revealed that in the glomeruli of Treg‐Exos^miR‐inhibition^ treated diabetic mice, the protective effects of Treg‐Exos on the mesangium, GBM, and foot processes were lost (Figure 4F–I). Compared with Treg‐Exos treated diabetic mice, Treg‐Exos^miR‐inhibition^ treated diabetic mice showed a significant increase in UACR (Figure 4J). IHC and WB results also revealed that the expression of SYNPO and WT1 in Treg‐Exos^miR‐inhibition^ treated diabetic mice was decreased (Figure 4K–N), the level of mitophagy was reduced (Figure 4O–Q; Figure S4C, Supporting Information), and apoptosis was increased (Figure 4R). In contrast, in vitro (Figure S5, Supporting Information) and in vivo experiments (Figure S6, Supporting Information) revealed that Treg‐Exos overexpressing miR‐218‐5p (Treg‐Exos^miR^) outperformed conventional Tregs‐Exos, restored the expression of podocyte markers, increased mitophagy, and relieved apoptosis and fibrotic response.

### 2.4 Tenascin‐C (TNC) is the Target gene of miR‐218‐5p

**Figure 5:**
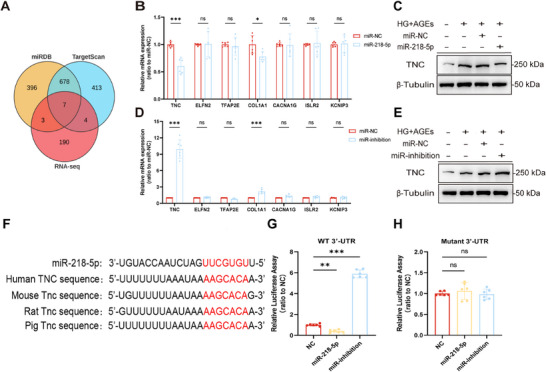
Treg‐Exos^miR^ targets regulate TNC. A) Venn diagram illustrating the intersection of RNA‐seq (upregulated DEGs in HG+AGEs groups with downregulated DEGs in Treg‐Exo^miR^ groups) and the potential target genes using two databases (TargetScan and miRDB). Quantitative PCR analysis of the expression of potential target genes in HPCs transfected with miR‐218‐5p (B) or miR‐218‐5p‐inhibition plasmids (D). *n* = 6 per group. Western blot analysis of TNC expression in HPCs transfected with miR‐218‐5p (C) or miR‐218‐5p‐inhibition plasmids (E). *n* = 3 per group. F) Sequence alignment of miR‐218‐5p with the 3′‐UTR of TNC. Luciferase activity of Human TNC 3′‐UTR in HPCs. HPCs were transfected with wild‐type (G) or mutant 3′‐UTR (H), together with miR‐218‐5p or miR‐218‐5p‐inhibition plasmids, and control plasmids. *n* = 6 per group. Images represent one representative experiment from three to six independent experiments. For all statistical plots, the data are presented as mean ± SD, and the distinct dots are represented as the individual values of 6 replicates. *p* values were calculated by a two‐tailed unpaired Student's *t* test, one‐way ANOVA, and Tukey's multiple comparison test. ns *p >0.05*, ^*^p < 0.05, ^**^p < 0.01, ^***^p < 0.001.

We performed RNA sequencing on human podocytes stimulated with a normal control, HG+AGEs, or co‐cultured with Treg‐Exos^miR^ and performed Kyoto Encyclopedia of Genes and Genomes (KEGG) analysis of the differentially expressed genes (DEGs) (Figure S7A–D, Supporting Information). Genes targeted by miR‐218‐5p were predicted using the bioinformatics websites TargetScan (https://www.targetscan.org/vert_80/) and miRDB (https://mirdb.org/). The downregulated DEGs in the HG+AGEs group and the upregulated DEGs in the Treg‐Exos group were compared and intersected. Seven intersecting genes were identified (**Figure** **5**A). After validation by qPCR and WB, miR‐218‐5p was found to have the most significant regulatory effect on TNC (Figure 5B–E). The 3′‐UTR of TNC contains a conserved binding site for miR‐218‐5p at positions 606–613. This conserved binding site exists not only in humans but also in other mammals, such as mice, rats, and pigs (Figure 5F). We speculate that TNC is a possible target gene of miR‐218‐5p.

We also performed KEGG enrichment analysis of the intersecting genes (Figure S7E, Supporting Information) and found that 13 genes were enriched mainly in the Toll‐like receptor pathway, such as TLR4, TLR3, and TLR1. We performed a heatmap analysis of the genes (Figure S7F, Supporting Information). These results indicated that miR‐218‐5p may reduce podocyte injury by targeting TNC and regulating the Toll‐like receptor signalling pathway. To further verify whether miR‐218‐5p can directly target TNC, a dual‐luciferase reporter assay was performed, and the results revealed that the luciferase reporter activity of the WT TNC 3′‐UTR was significantly inhibited by miR‐218‐5p and significantly increased after miR‐218‐5p inhibition (Figure 5G). The luciferase reporter gene activity of the Mutant TNC 3′‐UTR did not change significantly; that is, the mutated TNC was not regulated by miR‐218‐5p (Figure 5H).

### 2.5 TNC/SRC/FUNDC1 is a Key Signalling Pathway Through Which Treg‐Exos Exert Their Function

To confirm that TNC is a key molecule involved in the function of Treg‐Exos, we performed in vivo and in vitro functional rescue experiments. TNC protein expression levels were significantly increased in podocytes stimulated with HG+AGEs and in the kidney cortex of diabetic mice, but decreased after treatment with Treg‐Exos^miR^ (Figure S8A,B, Supporting Information).

**Figure 6:**
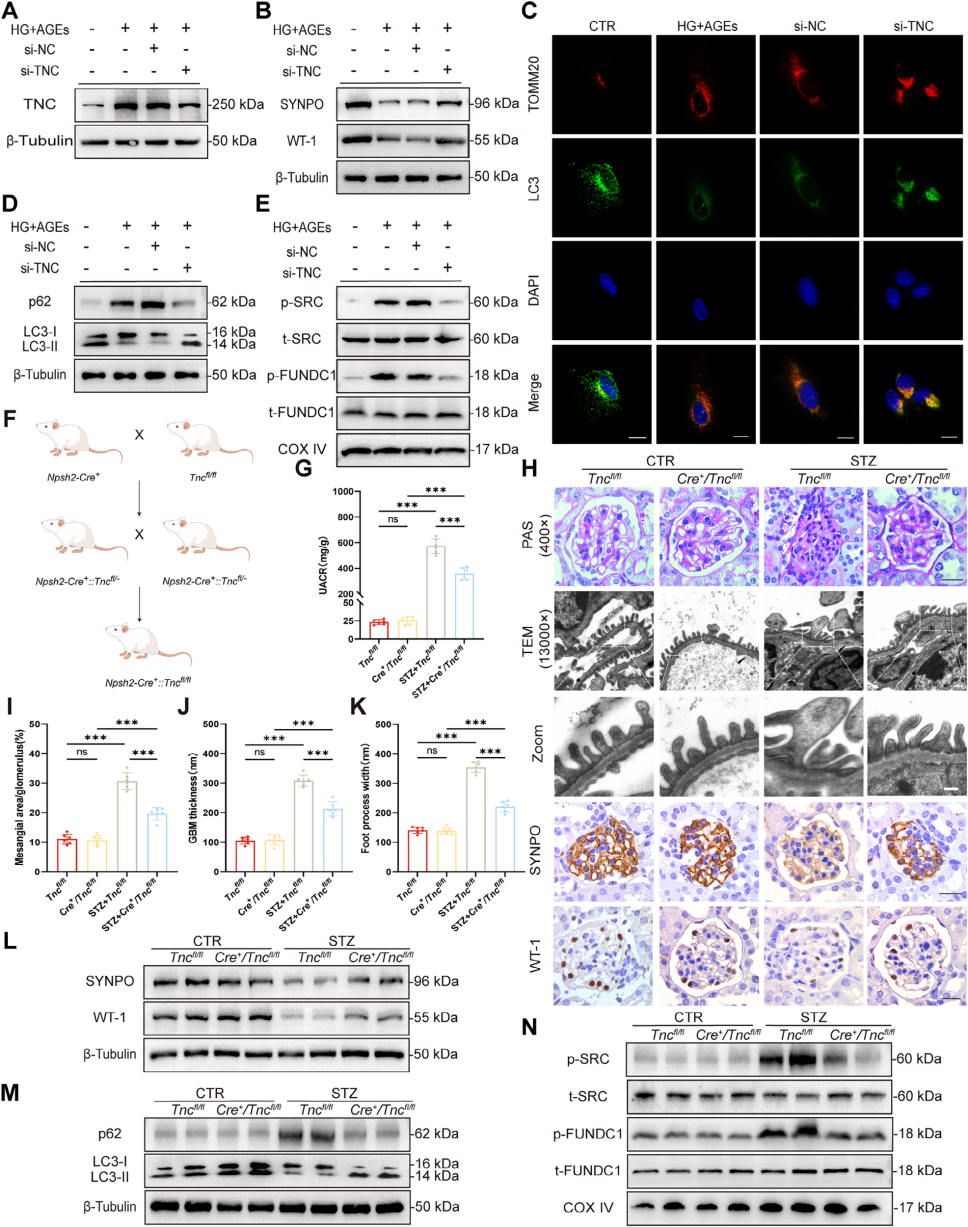
TNC knockdown alleviated podocyte injury both in vivo and in vitro. Western blot analysis of TNC **A**), SYNPO, and WT‐1 **B**) in HPCs transfected with si‐TNC for 48 h. *n* = 3 per group. **C**) Immunofluorescence staining of TOMM20 and LC3 expression in HPCs transfected with si‐TNC for 48 h. Scale bar, 20 nm. *n* = 3 per group. Western blot analysis of p62 expression and LC3 conversion **D**) and p‐SRC, t‐SRC, p‐FUNDC1, and t‐FUNDC1 (**E**) expression in HPCs transfected with si‐TNC for 48 h. *n* = 3 per group. F) Experimental scheme for generating *Nphs2‐Cre^+^/Tnc^fl/fl^* mice. G) Urinary albumin‐to‐creatinine ratios in *Nphs2‐Cre^+^/Tnc^fl/fl^* diabetic mice. *n* = 6 per group. H) Morphological examination of glomerular changes using PAS staining, TEM and IHC of SYNPO and WT‐1 in *Nphs2‐Cre^+^/Tnc^fl/fl^* diabetic mice by PAS staining, TEM, and IHC. Scale bar, 20 µm (PAS), 250 nm (TEM), 25 nm (Zoom), 20 µm (IHC), *n* = 6 per group. I) Quantification of the mesangial area in *Nphs2‐Cre^+^/Tnc^fl/fl^* diabetic mice. *n* = 6 per group. J) Quantification of GBM thickness in *Nphs2‐Cre^+^/Tnc^fl/fl^* diabetic mice. *n* = 6 per group. K) Mean width of podocyte foot processes in *Nphs2‐Cre^+^/Tnc^fl/fl^* diabetic mice. *n* = 6 per group. Western blot analysis of SYNPO, WT‐1 (L), p62, LC3 conversion (M), p‐SRC, t‐SRC, p‐FUNDC1, and t‐FUNDC1 (N) expression in the renal cortex of *Nphs2‐Cre^+^/Tnc^fl/fl^* diabetic mice. *n* = 3 per group. Images represent one representative experiment from three to six independent experiments. For all statistical plots, the data are presented as mean ± SD, and the distinct dots represent as the individual values of 6 replicates. *p* values were calculated by one‐way ANOVA and Tukey's multiple comparison test. ns *p >0.05*, ^***^p < 0.001.

In podocytes stimulated with HG+AGEs, TNC expression was reduced by si‐TNC (**Figure** **6**A), and the protein expression of SYNPO and WT‐1 was increased (Figure 6B). In addition, the level of mitophagy increased (Figure 6C–E), and apoptosis (Figure S9A, Supporting Information) decreased. For in vivo experiments, we constructed podocyte‐specific TNC knockout (*Nphs2‐Cre^+^/Tnc^fl/fl^* ^)^ mice (Figure 6F; Figure S10A–D, Supporting Information). *Nphs2‐Cre^+^/Tnc^fl/fl^* diabetic mice exhibited lower UACR (Figure 6G), alleviated podocyte injury, and reduced kidney lesion progression (Figure 6H–N; Figure S10B, Supporting Information). In addition, we overexpressed TNC in human podocytes and diabetic mice (Figure S11A,C, Supporting Information) and found that TNC could not directly injure podocytes (Figure S11B,D, Supporting Information).

**Figure 7:**
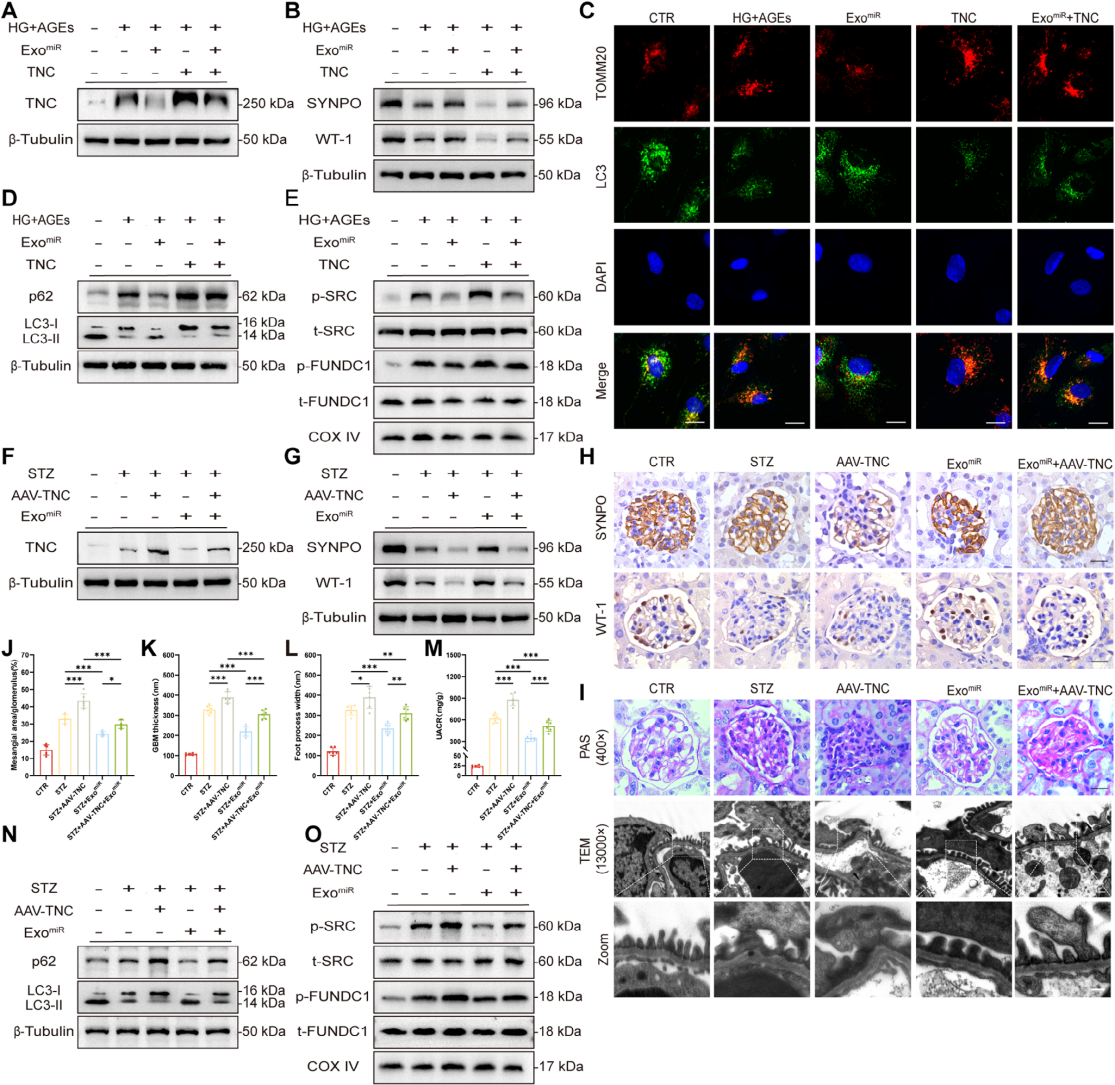
miR‐218‐5p regulates mitophagy by targeting TNC. Western blot analysis of TNC A), SYNPO, and WT‐1 B) expression in HPCs treated with Treg‐Exo^miR^ or/and transfected with TNC plasmids for 48 h. *n* = 3 per group. C) Immunofluorescence staining images of TOMM20 and LC3 expression in HPCs treated with Treg‐Exo^miR^ or/and transfected with TNC plasmids for 48 h. Scale bar,20 nm. *n* = 3 per group. Western blot analysis of p62 expression and LC3 conversion D) and p‐SRC, t‐SRC, p‐FUNDC1, and t‐FUNDC1 E) expression in HPCs treated with Treg‐Exo^miR^ and/or transfected with TNC plasmids for 48 h. *n* = 3 per group. Western blot analysis of TNC F), SYNPO, and WT‐1 G) expression in diabetic mice injected with Treg‐Exo^miR^ or/and AAV‐TNC. *n* = 3 per group. H) IHC analysis of SYNPO and WT‐1 expression in the glomeruli of diabetic mice injected with Treg‐Exo^miR^ and/or AAV‐TNC. Scale bar, 20 µm. *n* = 6 per group. I) Morphological examination of glomerular changes using PAS staining and TEM analysis in diabetic mice injected with Treg‐Exo^miR^ or/and AAV‐TNC. Scale bar, 20 µm (PAS), 250 nm (TEM), 25 nm (Zoom). *n* = 6 per group. J) Quantification of the mesangial area in diabetic mice injected with Treg‐Exo^miR^ or/and AAV‐TNC. n = 6 per group. K) Quantification of GBM thickness in diabetic mice injected with Treg‐Exo^miR^ or/and AAV‐TNC. *n* = 6 per group. L) Mean width of the podocyte foot processes in diabetic mice injected with Treg‐Exo^miR^ or/and AAV‐TNC. *n* = 6 per group. M) Urinary albumin‐to‐creatinine ratios in diabetic mice injected with Treg‐Exo^miR^ or/and AAV‐TNC. *n* = 6 per group. Western blot analysis of p62 expression, LC3 conversion N), p‐SRC, t‐SRC, p‐FUNDC1, and t‐FUNDC1 expression O) in the renal cortex of diabetic mice injected with Treg‐Exo^miR^ and/or AAV‐TNC. *n* = 3 per group. Images represent one representative experiment from three to six independent experiments. For all statistical plots, the data are presented as mean ± SD, the distinct dots are represented as the individual values of 6 replicates. *p* values were calculated by one‐way ANOVA and Tukey's multiple comparison test. ^*^p < 0.05, ^**^p < 0.01, ^***^p < 0.001.

To verify that TNC is a key molecule through which Treg‐Exos exert their function, human podocytes were treated with Treg‐Exos^miRs^ and/or transfected with TNC plasmids (**Figure** **7**A; Figure S12A,B, Supporting Information). WB and IF results demonstrated that overexpression of TNC reversed the protection of Treg‐Exos^miR^, including the protein level of podocyte markers (Figure 7B), the states of mitophagy (Figure 7C–E), and apoptosis (Figure S12C, Supporting Information). The results of the in vivo experiments were similar. After AAV‐TNC was used to induce high TNC expression in podocytes of diabetic mice, podocyte damage and kidney apoptosis were exacerbated. These injuries were significantly relieved after Treg‐Exos^miR^ treatment (Figure 7F–O; Figure S12D, Supporting Information).

Previous studies have shown that TNC is an endogenous agonist of TLR4.^[^ ^25^, ^26^ ^]^ We confirmed that the level of TLR4 was regulated by overexpression or knockdown of TNC (Figure S13A,B, Supporting Information). In addition, co‐immunoprecipitation (Co‐IP) experiments showed that TLR4 interacted with TNC (Figure S13C,D, Supporting Information). Human podocytes were transfected with si‐TNC and/or TLR4 (Figure S13E, Supporting Information). Based on the WB results, we believe that TLR4 overexpression reversed the protective effects of si‐TNC, including the protein levels of podocyte markers (Figure S13F, Supporting Information) and the state of mitophagy (Figure S13G,H, Supporting Information).

It has been reported that TLR4 can activate the phosphorylation of the SRC family to participate in various physiological and pathological activities. For example, TLR4 induces the phosphorylation of SRC Tyr 418 and is involved in the progression of atherosclerosis.^[^ ^27^ ^]^ To explore the relationship between TLR4 and SRC in human podocytes, we found that SRC phosphorylation was regulated by the overexpression or knockdown of TLR4 (Figure S14A,B, Supporting Information). After TLR4‐OE plasmids were used to induce high TLR4 expression in human podocytes, damage to podocytes and mitophagy were exacerbated. These injuries were significantly relieved after being treated with PP2, a SRC‐family inhibitor (Figure S14C–F, Supporting Information). Studies have also shown that in DKD, the phosphorylation of SRC Tyr416 can lead to the phosphorylation of FUNDC1 Tyr 18, thus reducing the recruitment of LC3II to mitochondria and thereby inhibiting the development of mitophagy in DKD podocytes.^[^ ^28^ ^]^

### 2.6 Engineered RGD‐Treg‐Exos Reduce Podocyte Injury More Effectively

**Figure 8:**
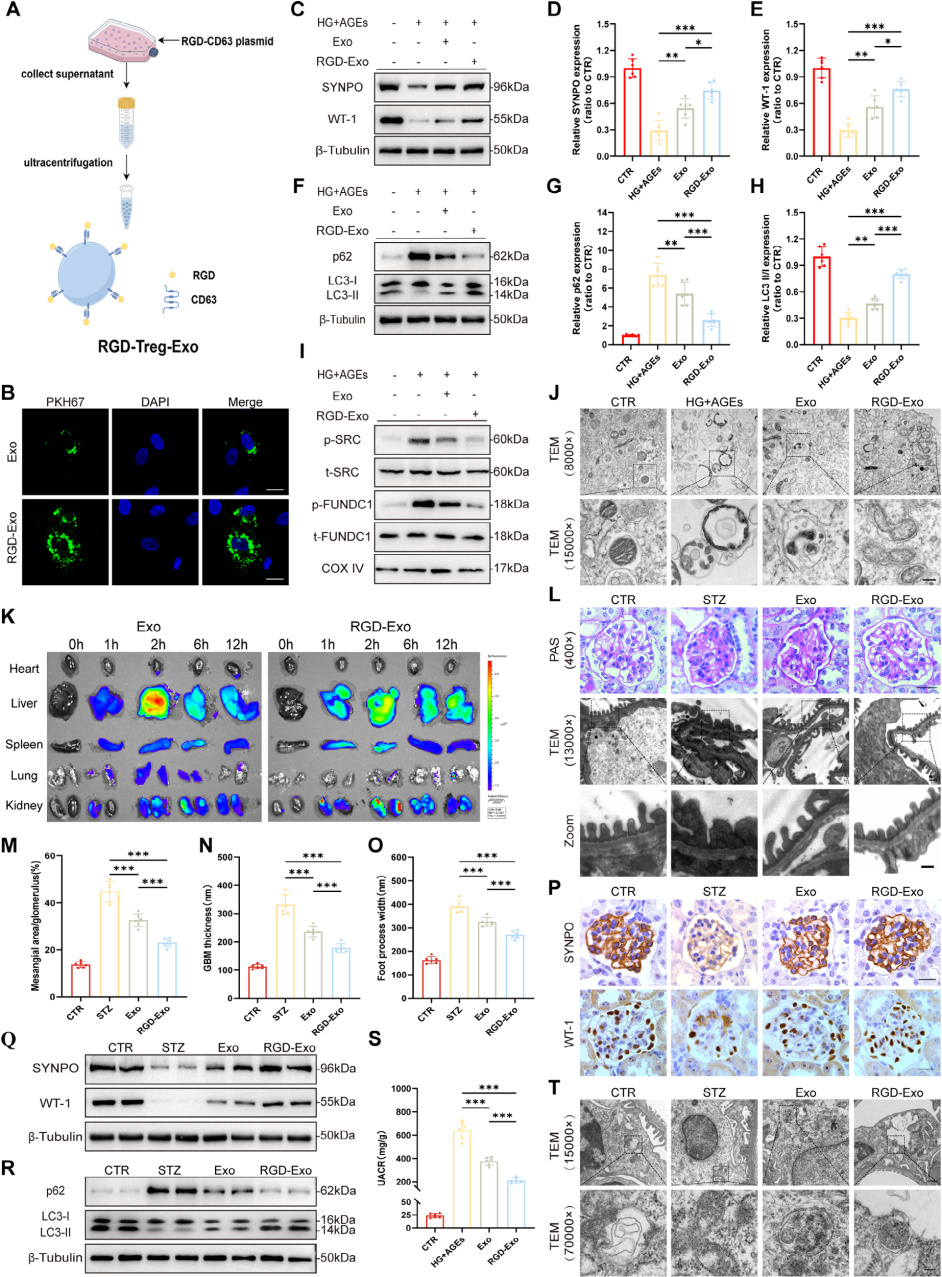
RGD‐Treg‐Exos alleviated podocyte injury more efficiently in vitro and in vivo. A) Schematic diagram of the synthesis of RGD‐Treg‐Exo. B) Fluorescence staining images of PKH67‐labeled RGD‐Treg‐Exos ingested by HPCs after 24 h of incubation. C) Western blot analysis of SYNPO, WT‐1 expression in HPCs treated with Treg‐Exos or RGD‐Treg‐Exos for 48 h. *n* = 6 per group. Quantitative analysis of the relative level of SYNPO (D) and WT‐1 (E) in (C). F) Western blot analysis of p62 expression and LC3 conversion in HPCs treated with Treg‐Exos or RGD‐Treg‐Exos for 48 h. *n* = 6 per group. Quantitative analysis of the relative level of p62 (G) and LC3 II/I (H) in (F). I) Western blot analysis of p‐SRC, t‐SRC, p‐FUNDC1and t‐FUNDC expression in HPCs treated with Treg‐Exos or RGD‐Treg‐Exos for 48 h. *n* = 6 per group. J) Morphological examination of mitochondria changes using TEM in HPCs treated with Treg‐Exos or RGD‐Treg‐Exos for 48 h. Scale bar, 4 µm (8000×), 2 µm (15000×), *n* = 3 per group. K) Fluorescence images of isolated organs from diabetic mice injected with DiR‐labeled Treg‐Exos or RGD‐Treg‐Exos. *n* = 3 per group. L) Morphological examination of glomerular changes by PAS staining and TEM in diabetic mice injected with Treg‐Exos or RGD‐Treg‐Exos. Scale bar, 20 µm (PAS), 250 nm (TEM), 25 nm (Zoom). *n* = 6 per group. M) Quantification of mesangial area in diabetic mice injected with Treg‐Exos or RGD‐Treg‐Exos. *n* = 6 per group. N) Quantification of GBM thickness in diabetic mice injected with Treg‐Exos or RGD‐Treg‐Exos. *n* = 6 per group. O) Mean width of podocyte foot processes in diabetic mice injected with Treg‐Exos or RGD‐Treg‐Exos. *n* = 6 per group. P) IHC analysis of SYNPO and WT‐1 expression in glomeruli of diabetic mice injected with Treg‐Exos or RGD‐Treg‐Exos. Scale bar, 20 µm. n = 3 per group. Western blot analysis of SYNPO, WT‐1 (Q), p62 expression, and LC3 conversion (R) in the renal cortex of diabetic mice injected with Treg‐Exos or RGD‐Treg‐Exos. *n* = 3 per group. S) Urinary albumin‐to‐creatinine ratios in diabetic mice injected with Treg‐Exos or RGD‐Treg‐Exos. *n* = 6 per group. T) Morphological examination of mitochondria changes using TEM in diabetic mice injected with Treg‐Exos or RGD‐Treg‐Exos. Scale bar, 5 µm (15000×), 1 µm (70000×), *n* = 3 per group. Images represent one representative experiment from three to six independent experiments. For all statistical plots, the data are presented as mean ± SD, and the distinct dots are represented as the individual values of 6 replicates. *p* values were calculated by one‐way ANOVA and Tukey's multiple comparison test. ^*^p < 0.05, ^**^p < 0.01, ^***^p < 0.001.

To achieve targeted delivery of Treg‐Exos to podocytes, we used engineering technology to load RGD‐Flag onto the exosome membrane protein CD63 (**Figure** **8**A). The engineering of RGD did not significantly affect the structure of the exosomes (Figure S15A–C, Supporting Information).

Our previous studies demonstrated that ITGAV was highly expressed in glomeruli and that targeted delivery was achieved through RGD peptide‐coupled compounds. ITGAV knockdown significantly reduced the uptake of RGD‐Treg‐Exos by podocytes (Figure S15D–F, Supporting Information). Laser scanning confocal microscopy (LSCM) that RGD‐Treg‐Exos were more effectively taken up by human podocytes than Treg‐Exos (Figure 8B). In addition, RGD‐Treg‐Exos significantly reduced podocyte injury (Figure 8C–E), restored mitochondrial morphology and mitophagy levels (Figure 8F–J; Figure S16A–C, Supporting Information), and attenuated apoptosis (Figure S16D, Supporting Information).

Similarly, in vivo experiments confirmed that RGD can increase the targeted uptake of exosomes. We generated podocyte‐specific IGTAV knockout mice (*Nphs2‐Cre^+^/Itgav^fl/fl^*) (Figure S17A–C, Supporting Information), confirming that integrin αvβ3 can mediate the uptake of RGD‐Exos by podocytes (Figure S17D, Supporting Information). To explore the targeting of RGD‐Treg‐Exos, we performed In Vivo Imaging System (veterinary imaging technology) (IVIS), and the results revealed that Treg‐Exos was mostly aggregated in the liver and had also been observed in the kidneys, spleen and lungs, whereas RGD‐Treg‐Exos was found to accumulate more in the kidneys, and decreased over time after reaching a peak level (Figure 8K). Cryosection of kidney tissues confirmed that RGD‐Treg‐Exos accumulated in the renal podocytes (Figure S17F, Supporting Information). Compared with the Treg‐Exos group, the widening of the glomerular mesentery, thickening of the basement membrane, and increase in foot process fusion in the RGD‐Treg‐Exos group were more significant (Figure 8L–O). IHC and WB results revealed that the expression of SYNPO and WT1 in podocytes in the RGD‐Treg‐Exos group increased (Figure 8P,Q). Furthermore, we also found that RGD‐Treg‐Exos significantly reduced UACR (Figure 8S), and that mitochondrial morphology and mitophagy levels were restored (Figure 8R,T; Figure S18A–C, Supporting Information). Moreover, apoptosis (Figure S17D, Supporting Information) was significantly reduced in the RGD‐Treg‐Exos group. In addition, we assessed the sustained effect and found that the fibrotic response (Figure S19A–C, Supporting Information) and serum Scr, BUN, ALT, and TBil levels were significantly reduced at 8 weeks after injection with Treg‐Exos or RGD‐Treg‐Exos.

## 3 Discussion

Podocyte injury or loss is a key event in the early stages of DKD, but currently, there is no specific treatment strategy targeting podocytes. Mitochondria are key organelles for energy generation and the maintenance of cellular homeostasis, especially in tissues with high energy demands, such as the glomeruli.^[^ ^29^, ^30^, ^31^ ^]^ In this study, we observed that mitophagy in the cytoplasm of podocytes was significantly inhibited in the DKD microenvironment and that many aggregated, damaged mitochondria could not be effectively removed. Therefore, restoring mitophagy may be a potential strategy for the treatment of podocyte injury in DKD.

Tregs are a special subgroup of CD4+ T cells and are considered important regulators of the maintenance of immune self‐tolerance and immune homeostasis,^[^ ^32^ ^]^ which are characterized by high expression of CD25 and the expression of the specific transcription factor forkhead box protein P3.^[^ ^33^ ^]^ As a novel cell‐free therapy, Treg‐Exos pose no risk of Treg phenotype switching and easily cross barrier structures, such as the blood‐brain barrier and glomerular filtration barrier. They have good potential for inhibiting transplantation rejection and drug transport.^[^ ^34^, ^35^ ^]^ Few studies have investigated the relationship between Tregs and their exosomes and kidney diseases. Previous studies have reported that proteinuria in patients with diabetes was significantly negatively correlated with the level of Tregs.^[^ ^36^ ^]^ Tregs can improve insulin resistance and DKD in db/db mice by suppressing inflammation and inhibiting CD8+ T cell infiltration in the kidneys.^[^ ^22^ ^]^ Studies have also shown that Treg‐Exos can inhibit T‐cell proliferation, delay acute rejection, and significantly prolong the survival of rats after kidney transplantation.^[^ ^37^ ^]^ However, the protective effects of Tregs and their exosomes on intrinsic kidney cells, especially podocytes, remain unclear. In this study, we confirmed that Treg‐Exos restored the level of mitophagy in podocytes and reduced podocyte injury both in vivo and in vitro.

miRNAs play important roles in podocyte injury. Abnormal miRNA expression levels in podocytes can cause mitochondrial dysfunction, which can lead to podocyte injury and disruption of kidney function. For example, the miR‐30 family induces podocyte cytoskeleton damage by regulating the expression of proteins, such as TRPC6.^[^ ^38^, ^39^ ^]^ In this study, we performed miRNA sequencing of human podocytes and Treg‐Exos and revealed that miR‐218‐5p mediates the mechanism by which Treg‐Exos protect podocytes. At present, there are few studies on miR‐218‐5p in kidney disease, and most studies have focused on its role in cancer.^[^ ^40^ ^]^ A previous study indicated that, after ischemic preconditioning in vitro, the level of miR‐218‐5p increased in human kidney‐derived endothelial cells, which relieved injury in endothelial cells.^[^ ^41^, ^42^ ^]^ Our results indicate that Treg‐Exos increase mitophagy and reduce podocyte injury in DKD through the delivery of miR‐218‐5p.

We performed RNA sequencing and identified the target gene TNC. TNC participates in various pathophysiological processes by binding to cell surface receptors, such as integrin, Toll‐like, and epidermal growth factor (EGF) receptors, and activating their downstream signalling pathways.^[^ ^43^, ^44^, ^45^, ^46^ ^]^ Moreover, we found that the gene sets obtained by sequencing were mainly enriched in the Toll‐like receptor pathway. This is consistent with the conclusion that TNC is an endogenous agonist of TLR4.^[^ ^25^, ^26^ ^]^ Next, we constructed *Nphs2‐Cre^+^/Tnc^fl/fl^* mice and found that podocyte damage and kidney lesions were significantly reduced in the TNC knockout mice. Moreover, TNC overexpression in podocytes aggravated podocyte injury. In contrast, after the administration of Treg‐Exos^miR^, podocyte damage was significantly reduced and disease progression was attenuated. Therefore, we believe that TNC is a key molecule through which Treg‐Exos^miR^ functions. SRC is an important member of the SRC family of kinases, which are widely expressed in the cytoplasm of different cells and are mainly involved in the signal transduction process in cells.^[^ ^47^, ^48^ ^]^ Previous studies and our results have shown that TLR4 improves SRC Tyr 418 phosphorylation and is involved in atherosclerosis progression of atherosclerosis.^[^ ^27^ ^]^ FUNDC1 is a mitochondrial outer‐membrane protein. In DKD, the phosphorylation of SRC Tyr416 can lead to the inactivation of FUNDC1 Tyr18 phosphorylation, thus reducing the recruitment of LC3II to the mitochondria, thereby inhibiting the development of mitophagy in DKD podocytes.^[^ ^28^ ^]^ We found that TLR4 obviously phosphorylates SRC Tyr416 to mediate the downstream SRC/FUNDC1 signalling pathway and inhibits the clearance of damaged mitochondria.

Because the specificity degree of exosome targeting is not high, to increase the enrichment of exosomes in specific tissues and organs, we modified the surface of exosomes to increase their targeting ability. The membranes of exosomes are mainly composed of transmembrane proteins, such as LAMP‐2B, CD63, and CD9. These membrane proteins can be modified through genetic engineering to produce exosomes containing a target protein that can bind to the corresponding ligands to improve the specific targeting of exosomes.^[^ ^49^, ^50^ ^]^ We constructed an RGD‐CD63 plasmid and verified its target ability by ITGAV knockdown human podocytes and *Nphs2‐Cre^+^/Itgav^fl/fl^* mice. Subsequent in vivo and in vitro experiments revealed that RGD‐Treg‐Exos effectively delivered miR‐218‐5p to podocytes, restored mitophagy, and reduced podocyte injury.

In summary, this study revealed that Treg‐Exos restored the level of mitophagy in damaged podocytes and attenuated apoptosis and fibrotic response through the miR‐218‐5p/TNC/TLR4/SRC/FUNDC1 pathway. Engineered RGD‐Treg‐Exos effectively improved podocyte injury in DKD and represent a novel method for the treatment of DKD.

## 4 Experimental Section

### Human Samples

Six patients with DKD who underwent renal biopsy at the National Clinical Research Center of Kidney Diseases, Jiangsu Biobank of Clinical Resources, at Jinling Hospital affiliated with Nanjing University, were selected. The inclusion criteria were clinical indicators of DKD; three random urine tests within 3 to 6 months with UACR>30 mg g^−1^, eGFR<60 mL/min/1.73 m^2^; renal biopsy indicating advanced DKD glomerulosclerosis. The exclusion criteria were diabetes combined with NDKD; family history of kidney disease; hepatitis virus or human immunodeficiency virus infection; malignant tumors; and other diseases, such as those of the liver, heart, or hematopoietic system. As controls, blood samples were collected from 6 healthy volunteers with no known history of kidney disease. Renal tissues from nephrectomy patients (*n* = 6) with renal cancer were obtained from the kidney biobank of the center. Paracancerous renal tissues of patients who underwent nephrectomy did not show clinical features of renal dysfunction. Table S1 (Supporting Information) shows the demographic and clinical characteristics of patients and individuals in the control group. All study protocols involving human sample collection were approved by the Ethics Committee of the Affiliated Jinling Hospital, Medical School of Nanjing University (Document number 2023DZGZR‐100), and written informed consent was obtained from each subject before enrolment.

### Animals

Healthy male C57BL/6J mice (3‐4 weeks; body weight, 20 ± 2 g) were provided by GemPharmatech (Nanjing, China). *Itgav‐Flox* (NM‐CKO‐200110), *Tnc‐Flox* (NM‐CKO‐233220), and *Nphs2‐Cre* mice were obtained from Shanghai Model Organism Centre Co., Ltd. (Shanghai, China). Podocyte‐specific Tnc and Itgav knockout mice were generated by crossing *Nphs2‐Cre* mice with *Tnc‐Flox* and *Itgav‐Flox* mice (*Nphs2‐Cre^+^/Tnc^fl/fl^* and *Nphs2‐Cre^+^/Itgav^fl/ fl^*). Tail snips and PCR were performed at 2 weeks of age for genotyping. Age‐matched *Tnc^fl/fl^* and *Itgav^fl/fl^* mice were used as controls. All animals were housed under standard conditions (25 °C, 55% air humidity), with free access to food and water. All animal experiments were approved by the Institutional Animal Care and Ethics Committee of the Affiliated Jinling Hospital, Medical School of Nanjing University. (Document number 2023JLHGZRDWLS‐000114).

### Cell Lines

Immortalized human podocytes (HPCs) were provided by M. Salem (University of Bristol, Bristol, UK). HPCs were cultured in RPMI1640 medium (Gibco, Thermo Fisher Scientific, Waltham, Massachusetts, USA) with 10% exosome‐free serum (Oricell, San Francisco, California, USA), 10% fetal bovine serum (Gibco), 1% insulin‐transferrin‐selenium (ITS) (Gibco), 100 U mL^−1^ penicillin (Gibco) along 0.1 mg mL^−1^ streptomycin (Gibco) under suitable conditions (33 °C, 5% CO2). To induce differentiation, cells were cultured at 37 °C for 10 days and used in the experiments.

### Animal Model and Treatment

C57BL/6J, *Nphs2‐Cre^+^/Tnc^fl/fl^*, and *Tnc^fl/fl^* mice were fed a high‐fat diet (HFD) for 4 weeks. At eight weeks of age, mice were intraperitoneally injected with streptozotocin (STZ (Sigma‐Aldrich, St. Louis, MO, USA). Louis, USA) at a dose of 50 mg/(kg·d) for five consecutive days. The blood glucose levels were measured weekly. Modelling was considered successful when mice had a fasting blood glucose level higher than 16.6 mmol L^−1^ and identifiable proteinuria.^[^ ^51^ ^]^ All animals were randomly grouped at the time of use. The model mice were subsequently processed at 4 weeks and euthanized at 8 or 12 weeks, after which urine, blood, and kidney tissues were collected. The control group (CTR group) consisted of normal C57BL/6J mice that had not received any treatment. Our study examined both male and female animals, and similar findings were reported for both sexes.

To assess the efficacy of Treg‐Exos, diabetic mice were injected via the tail vein with 30 µg/d (≈3*10^9^ particles/d) Treg‐Exos; PBS was used as a control (*n* = 6). To assess the efficacy of Treg‐Exos^miR^ and Treg‐Exos^miR‐inhibition^, diabetic mice were injected with the same amount of Treg‐Exos^miR^ and Treg‐Exos^miR‐inhibition^ via the tail vein. Treg‐Exos^miR‐NC^ was used as control (*n* = 6). To examine the protective effect of TNC knockout on the kidneys, *Nphs2‐Cre^+^/Tnc^fl/fl^* and *Tnc^fl/fl^* mice were euthanized at 8 weeks, and urine, blood, and kidney tissues were collected (*n* = 6). To determine whether TNC is a key molecule for the function of Treg‐Exos^miR^, diabetic mice were injected via the tail vein with 10^9^ IFU/adenovirus (AAV‐Tnc) (HANBIO, Shanghai, China), and 30 µg/day Treg‐Exos^miR^, AAV‐Tnc, or Treg‐Exos^miR^ alone was injected as a vector control (*n* = 6). To assess the efficacy of Treg‐Exos, diabetic mice were injected with the same amount of Treg‐RGD‐Exos via the tail vein, and Treg‐Exos were used as controls (*n* = 6). To assess the fibrotic response of diabetic mice, Treg‐Exos and Treg‐RGD‐Exos were injected via the tail vein for 8 weeks, and PBS was used as a control (*n* = 6).

### Cell Culture and Treatment

Differentiated HPCs were incubated with 30 mm glucose (MCE, South Plainfield, New Jersey, USA) and 100 µg mL^−1^ AGEs (MCE) in a simulated diabetic environment for 24 or 48 h. The control group (CTR group) was cultured in complete medium without any treatment. To determine the mode of Treg function, HPCs were seeded in the upper chamber of Transwell plates (0.3 µm polycarbonate filter, Servicebio, Wuhan, China), and Tregs were seeded in the lower chambers of Transwell plates. Cocultures without transwell plates were used as control (*n* = 6). To investigate the role of Treg‐Exos, HPCs were treated with 5 µg mL^−1^ Treg‐Exos (≈5*10^3^ particles/cell) under HG+AGEs conditions, and free medium (Treg cell supernatant from which the exosomes were removed was mixed with RPMI 1640 complete medium at a ratio of 1:1) was used as a control (*n* = 6). For co‐culture, the cells were treated with 10 µM GW4869 (Sigma–Aldrich) for 24 h to inhibit the production of exosomes; the same volume of DMSO (Sigma–Aldrich) was used as a control (*n* = 6). To assess the efficacy of Treg‐Exos^miR^ and Treg‐Exos^miR‐inhibition^, equal amounts of Treg‐Exos^miR^ and Treg‐Exos^miR‐inhibition^ were used to treat HPCs under HG + AGEs conditions. Treg‐Exos^miR‐NC^ was used as a control (*n* = 6). To assess the protection role by TNC knockout, HPCs were treated with si‐TNC under HG+AGEs conditions; si‐NC served as a control (*n* = 6). To investigate whether TNC is a key molecule through which Treg‐Exos exert their functions, Treg‐Exos were used to treat TNC‐overexpressing HPCs under HG + AGEs conditions. HPCs overexpressing TNC alone or treated with Treg‐Exos^miR^ under HG + AGEs conditions were used as controls (*n* = 6). To investigate the interaction between TLR4 and TNC, TLR4 was used to treat TNC‐knockdown HPCs under HG + AGEs conditions. HPCs overexpressing TLR4 alone or si‐TNC under HG + AGEs conditions were used as controls (*n* = 6). To investigate the interaction between TLR4 and SRC, PP2 (MCE) was used to treat TLR4‐overexpressing HPCs under HG + AGEs conditions. HPCs overexpressing TLR4 alone or treated with PP2 under HG + AGEs conditions were used as controls (*n* = 6). To assess the targeting ability of RGD‐Treg‐Exos, HPCs were treated with si‐ITGAV under HG+AGEs conditions; si‐NC was used as a control (*n* = 6). To assess the efficacy of RGD‐Treg‐Exos, HPCs were treated with RGD‐Treg‐Exos under HG+AGEs conditions; Treg‐Exos were used as control (*n* = 6).

### In Vitro Culture and Identification of Tregs

Peripheral blood from healthy controls was isolated using a lymphocyte separation medium (Solarbio, Beijing, China) to generate single‐cell suspensions. A human CD4+CD25+ regulatory T cell isolation kit (Miltenyi Biotec, Bergisch Gladbach, Germany) was used to isolate CD4+CD25+Treg cells, according to the instruction manual.^[^ ^34^ ^]^ Briefly, the process was divided into two parts. First, non‐CD4+ T cell negative selection was performed, followed by positive selection of the obtained CD4+ T cells using CD25 magnetic beads. Under appropriate conditions (37 °C and 5% CO_2_), Tregs were supplemented with 25 µL/mL human CD3/CD28 activator (Stemcell, Vancouver City, Canada), 500 U mL^−1^ recombinant human cytokine IL‐2 (Beyotime, Shanghai, China), 10% human AB serum (PBM, Morrisville, Munich, Germany) and 100 U mL^−1^ penicillin along with 0.1 mg mL^−1^ streptomycin.^[^ ^11^ ^]^ After culturing in 10% serum with the exosomes removed, Treg cell supernatants were collected every two days for exosome isolation.

After washing, Tregs were resuspended in PBS, and FITC‐conjugated anti‐hu CD4, PE‐conjugated anti‐hu CD25, and APC‐conjugated anti‐hu CD127 antibodies (see Table S4 for details, Supporting Information) were added, followed by incubation at room temperature in the dark. The cells were assessed using a flow cytometer (BD Biosciences, San Jose, California, USA) and analyzed using FlowJo 10.8.

### Isolation and Identification of Exosomes

To remove cells, cell debris, and apoptotic bodies, Treg supernatants were centrifuged at 300 × g, 2000 × g and 10000 × g respectively for 10 min and then filtered through a 0.22 m filter (Millex‐GP) (MilliporeSigma, Darmstadt, Germany). Exosomes were obtained by centrifugation at 100 000 g for 1 h in an ultracentrifuge (Optima L‐100XP, Beckman Coulter, California, USA).^[^ ^52^ ^]^

Exosome morphology was observed using TEM (Hitachi, Tokyo, Japan). The diameter and number of exosomes were assessed using NTA (Particle Metrix; Karlsruhe, Germany). WB was used to investigate the presence of biomarkers on the exosome surface, including CD63, Alix, and TSG101, and calnexin was used as a negative control (see Table S3 for details, Supporting Information).

### Transfection

To overexpress or knock down miR‐218‐5p or overexpress RGD in Treg‐Exos, we used jetPRIME transfection reagent (Polyplus, Strasbourg, France) to transfect miR‐218‐5p, miR‐218‐5p‐inhibition, control plasmids (Gene Chem), and RGD‐CD63 along with control plasmids (Gene Chem, Shanghai, China) into Tregs according to the instruction manual.

To overexpress or knock down TNC, TLR4, or ITGAV in HPCs, we used the same method to transfer TNC overexpression (HANBIO), si‐TNC (Gene Script, Nanjing, China), TLR4 overexpression (HANBIO), si‐ITGAV (Gene Script), and control plasmids or si‐NC into the HPCs.

### Cellular Uptake Experiment

To determine whether the exosomes obtained by ultracentrifugation were biologically active and could be taken up by HPCs, we incubated exosomes with 100 µm PKH67 (BestBio, Shanghai, China) at 37 °C for 15 min, after which the excess dye was removed by washing and ultracentrifugation. Fluorescence‐labeled exosomes were incubated with HPCs for 24 or 48 h. Then, the cells were washed with PBS, fixed with 4% paraformaldehyde (Servicebio), permeabilized with 0.3% Triton X‐100 (Sigma–Aldrich), and incubated with DAPI (Beyotime) to visualize the nuclei. After the cells were mounted with an antifade reagent (Biosharp, Hefei, China), they were observed and recorded under a fluorescence microscope (Leica DM5000B Microscope, Wetzlar, Germany).

### Measurement of the Mitochondrial Membrane Potential (JC‐1)

The mitochondrial membrane potential (Δψm) of human podocytes was assessed using a JC‐1 probe (Yesen, Shanghai, China). Podocytes were incubated with 10 µL mL^−1^ JC‐1 staining solution at 37 °C for 20 min. After washing, images were obtained using a laser confocal scanning microscope (LSM710, ZEISS, Oberkochen, Germany). Red and green fluorescence intensities were quantified using Zeiss LSM image inspector software (ZEN). Red fluorescence represents JC‐1 aggregates, green fluorescence represents the monomeric form of JC‐1, and the ratio of red to green fluorescence intensity reflects Δψm.

### Urine ALB and Creatinine

Urine samples were collected at 8 weeks and centrifuged at 3000 × g for 10 min to remove precipitates in the urine. The levels of ALB and creatinine were measured using a mouse ALB ELISA kit (Elabscience) and a creatinine assay kit (Nanjing Jiancheng Bioengineering Institute, Nanjing, China), according to the manufacturer's instructions.

### Blood Glucose Testing

The mice were fasted overnight with normal access to drinking water. Fasting blood glucose concentrations of the mice were measured using a blood glycosometer (Yuwell, Shanghai, China) and blood glucose test strips (Yuwell).

### In Vivo Tracer and Uptake Experiments in Animals

To investigate the distribution of exosomes in mice and whether exosomes can be taken up by podocytes, we incubated PKH67 exosomes with 1 µM DiR (Yesen) at 37 °C for 15 min, after which excess dye was removed by washing. Fluorescence‐labeled exosomes were injected into mice through the tail vein, and fluorescence signals in the mice were evaluated using an IVIS imaging system (PerkinElmer, Waltham, USA). Frozen sections of kidney tissue were prepared at 4 weeks for immunofluorescence staining to analyze the uptake of exosomes by podocytes.

### TEM

Similar to our previous study,^[^ ^53^ ^]^ HPCs and 1 mm^3^ renal cortex tissue samples were fixed in 3.75% glutaraldehyde (Servicebio) and then fixed in 1% osmium tetroxide (Sigma‐Aldrich) in phosphate buffer. After dehydration, the samples were embedded in epoxy resin (Sigma–Aldrich), subjected to ultrathin sectioning at 70 nm, stained with 2% uranyl acetate‐lead citrate, and observed under a Hitachi 7500 electron microscope. To measure GBM thickness, foot process width, and mitochondrial morphology, the images were analyzed using a Gatan Microscope Suite 3.0 (AMETEK, Berwyn, Pennsylvania, USA).

### MicroRNA Sequencing Analysis

Total RNA was extracted from human podocytes and Treg‐Exos using TRIzol reagent. Polyacrylamide gel electrophoresis (PAGE) was used to select bands ranging from to 18–30 nt in length, and the small RNA was recovered. The 3′ adapter and the 5′ adapter were ligated, and small RNA with adapters ligated on both sides was subsequently subjected to reverse transcription and PCR. A band of ≈140 bp was recovered, purified via PAGE, and dissolved in EB solution to complete library construction. The constructed libraries underwent quality control and were sequenced using an Agilent 2100 and ABI StepOnePlus Real‐Time PCR System (Life Technologies, Thermo Fisher Scientific). Small RNA library construction and sequencing were performed at Genedenovo Biotechnology Co. Ltd. (Guangzhou, China). miRNA dynamic heatmaps were analyzed using OmicShare tools (https://www.omicshare.com/tools), and *P*<0.05 was considered a significantly differentially expressed miRNA.

### Fluorescence miRNA In Situ Hybridization

The fluorescence intensity of miR‐218‐5p in HPCs, mouse kidney tissues, and kidney biopsy samples from DKD patients was assessed using the miR‐218‐5p fluorescent probe following the protocol provided with the miRNA FISH kit (GenePharma, Shanghai, China). The cells were then stained with SYNPO, and the nuclei were labeled with DAPI for visualization.

### RNA Sequencing and Analysis

After total RNA from human podocytes was extracted using TRIzol, the enriched mRNA was reverse transcribed to generate double‐stranded cDNA. After the paired ends of the cDNA were repaired, adapters were added, and the cDNA was subsequently amplified via PCR to construct a library. To ensure sequencing quality, we used strict quality control to check the quality of library construction. The integrity of the RNA samples and the presence of DNA contamination were analyzed via agarose gel electrophoresis, RNA purity was determined using a NanoPhotometer spectrophotometer, RNA concentration was precisely quantified using a Qubit 2.0‐fluorometer, and RNA integrity was accurately assessed using an Agilent 2100 bioanalyzer. cDNA library construction and sequencing were performed at Genedenovo Biotechnology Co. Ltd. DESeq2 software was used to identify differentially expressed genes in the RNA sequencing data between the two groups. Genes with P<0.05 were considered significantly differentially expressed genes.

### Dual‐Luciferase Reporter Assay

The jetPRIME transfection reagent was used according to the manufacturer's instructions. HPCs were transfected with luciferase 3′‐UTR reporter gene constructs (TNC WT 3′‐UTR and TNC MUT 3′‐UTR) (HANBIO), miRNAs (miR‐NC, miR‐218‐5p, and miR‐218‐5p‐inhibition), and Renilla luciferase (HANBIO). After 24 h of transfection, the luciferase activity of the cells was measured using a Dual‐Luciferase Assay Kit (HANBIO) and a full‐wavelength multipurpose microplate reader (SpectraMax M5, Molecular Devices, San Jose, California, USA). The values for firefly luciferase were normalized to those for Renilla luciferase.

### Co‐Immunoprecipitation Assay

HPCs were lysed in IP lysis buffer according to the instructions of the immunoprecipitation kit (Proteintech, Wuhan, China). A small amount of lysate was used for WB assays. The remaining lysate was subjected to antigen‐antibody binding to form antigen‐antibody complexes. After immunoprecipitation, the protein A/G beads were precipitated by centrifugation, and the supernatant was discarded. The antigen‐antibody complexes were eluted after centrifugation and washed with lysis buffer. The presence of target antigens was assessed by WB.

### Immunohistochemical (IHC) Analysis and PAS Staining

Mouse kidney tissues were fixed in 4% paraformaldehyde buffer, embedded in paraffin, and sectioned (4 µm). PAS staining was performed according to the instructions of a staining kit (Servicebio). IHC was performed as described previously.^[^ ^51^ ^]^ After deparaffinization, antigen retrieval, and permeabilization, the tissue sections were blocked at room temperature, incubated with primary antibodies at 4 °C overnight (the antibodies used are listed in Table S5, Supporting Information), and incubated with goat anti‐rabbit/mouse IgG H&L antibodies (Proteintech). DAB substrate (Proteintech) was added to the sections, which were then incubated at room temperature for 1 min. The sections were counterstained with hematoxylin (Servicebio) for 1 min at room temperature. Histological images were obtained under a light microscope.

### Immunofluorescence Analysis

Immunofluorescence analysis of the kidney tissue sections was performed as previously reported.^[^ ^53^ ^]^ Briefly, the sections were blocked and incubated with primary antibodies at 4 °C overnight. After washing, sections were incubated with the corresponding fluorescent secondary antibodies at room temperature. The nuclei were stained with DAPI. The sections were mounted using an an antifade reagent for observation. The fluorescent antibodies used are listed in Table S5 (Supporting Information). Images were captured using a fluorescence microscope and processed using software.

### RNA Extraction, Reverse Transcription and RT‐qPCR

Total RNA was extracted using the TRIzol reagent (Invitrogen, Carlsbad, California, USA). The extracted RNA was reverse‐transcribed into cDNA using PrimeScript RT Master Mix (Perfect Real Time) (Takara, Kyoto, Japan). Real‐time quantitative RT‐PCR was performed using SYBR qPCR Master Mix (Vazyme, Nanjing, China). To determine the miRNA levels, stem‐loop RT‐qPCR was performed using miRNA‐specific primers. Briefly, cDNA was obtained using a MicroRNA First‐Strand Gene Synthesis Kit (stem‐loop method) (Accurate Biology, Changsha, China). Real‐time PCR was performed using SYBR Green Pro Taq HS Premix qPCR Kit (Accurate Biology). The mRNA and miRNA levels of the target genes were analyzed using the Applied Biosystems QuantStudio3 Real‐time PCR System (Thermo Fisher Scientific). Tables S1 and S2 (Supporting Information) list the specific primers used for the target genes in this study. The levels of the housekeeping genes GAPDH and U6 were used as the internal controls. The samples were normalized via the 2^−ΔΔCT^ method and analyzed for changes in gene expression.

### Western Blot Analysis

The samples were homogenized in RIPA buffer (Roche, Basel, Switzerland) containing protease and phosphatase inhibitors, and the supernatant (total protein) was obtained after centrifugation. Mitochondrial proteins were obtained by differential centrifugation using a mitochondrial isolation kit (BestBio). After protein concentration was determined using a BCA protein concentration assay kit (Beyotime), WB analysis was performed. Briefly, after protein samples were extracted and separated by SDS‐PAGE, the proteins were electrophoretically transferred to a PVDF membrane (Bio‐Rad, Hercules, USA). After blocking, the membrane was incubated with a primary antibody at 4 °C overnight, washed, incubated with a secondary antibody, and visualized following incubation in an ECL luminescence solution (NCM Biotech, Suzhou, China). All the primary and secondary antibodies used are listed in Tables S4 and S5 (Supporting Information), respectively. Images were collected using a TANON 5200 fully automatic chemiluminescence image analysis system (TANON, Shanghai, China), and band intensities were analyzed using images.

### Statistical Analysis

The data were expressed as mean ± SD or SEMs. Statistical analyses were performed using IBM SPSS (version 26, Statistical Packages Corp., Armonk, NY, USA). Plots were generated using GraphPad Prism (version 9.0, GraphPad Software, USA). The normality of the data distribution was assessed using the Kolmogorov‐Smirnov test. Comparisons of normally distributed data between the two groups were performed using a two‐tailed *t*‐test. Comparisons among multiple groups were performed using one‐way analysis of variance (ANOVA), followed by Tukey's test to identify differences. Significance was defined as ns P>0.05, ^*^P<0.05, ^**^P<0.01, and ^***^ P<0.001. The sample size for each experiment was based on the experience of our laboratory in previous studies using DKD animal models and gene knockout mice.

## Conflict of Interest

The authors declare no conflict of interest.

## Author Contributions

Z.C.G. and S.H.G. designed and performed experiments. Z.G.C., Z.C.G., Z.Y.W., J. L. C., A. P. D.,F. X., and Q.E.W. analyzed the data. W.S.Q., C.H.Z., Z.H.L., and H.B. drafted and revised the manuscript. Z.H.L. and H.B. conceived, designed, and supervised the study. Z.C.G. and S.H.G. contributed equally to this work. All the authors approved the final version of the manuscript.

## Supporting information



Supporting Information

## Data Availability

The data that support the findings of this study are available in the supplementary material of this article.
