# Supplementary material for: Engineered RGD‐Treg‐Exos Targeted Delivery of miR‐218‐5p to Activate Mitophagy and Attenuate Podocyte Injury in Diabetic Kidney Disease
Source: Adv Sci (Weinh). 2025 Aug 19;12(37):e12034. doi: 10.1002/advs.202412034 (PMC12499504; doi:10.1002/advs.202412034)
Supplement: Supplementary file 1 — Supporting Information [file ADVS-12-e12034-s001.docx]

Supplementary Materials For

**Engineered RGD-Treg-Exos targeted delivery of miR-218-5p to activate mitophagy and attenuate podocyte injury in diabetic kidney disease**

Zhaochen Guo^1#^, Shaohui Gao^1#^, Ziyue Wang^1^, Zige Chen^1^, Jinglei Chen^1^, Aiping Duan^1^, Feng Xu^3^, Qinger Wang^1^, Weisong Qin^1^, Caihong Zeng^1^, Zhihong Liu^1*^ & Hao Bao^1,2*^

Correspondence to Hao Bao (bhao@nju.edu.cn) or

Zhihong Liu (liuzhihong@nju.edu.cn)

**This file includes:**

Supplementary Figures 1-19

Supplementary Tables 1-5


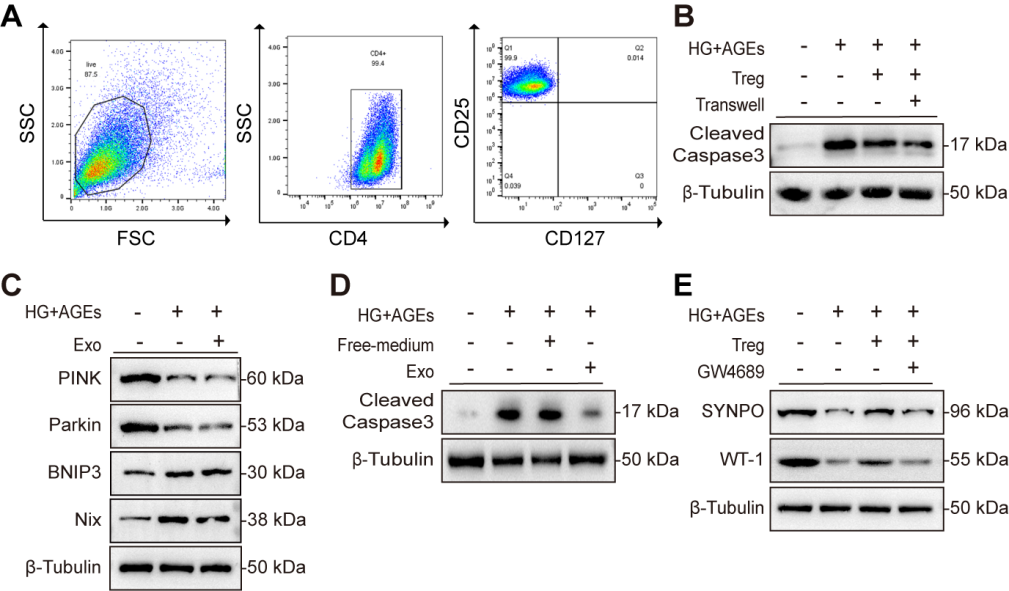


**Figure S1 Tregs and Treg-Exos alleviate podocyte injury induced by HG+AGEs.**

A) Flow cytometry sorting of CD4^+^CD25^+^CD127^-/low^ Treg-Exos. n=3 per group. B) Western blot analysis of cleaved caspase3 expression in HPCs after co-culturing with Tregs for 48 hours. n=3 per group. C) Western blot analysis of PINK, Parkin, BNIP3 and Nix expression in HPCs treated with Treg-Exos for 48 hours. n=3 per group. D) Western blot analysis of cleaved caspase3 expression in HPCs treated with Free medium or Treg-Exos for 48 hours. n=3 per group. E) Western blot analysis of SYNPO and WT-1expression in HPCs after co-culturing with Tregs using GW4869 or not for 48 hours. n=3 per group. Images are from one representative experiment from 3 independent experiments.


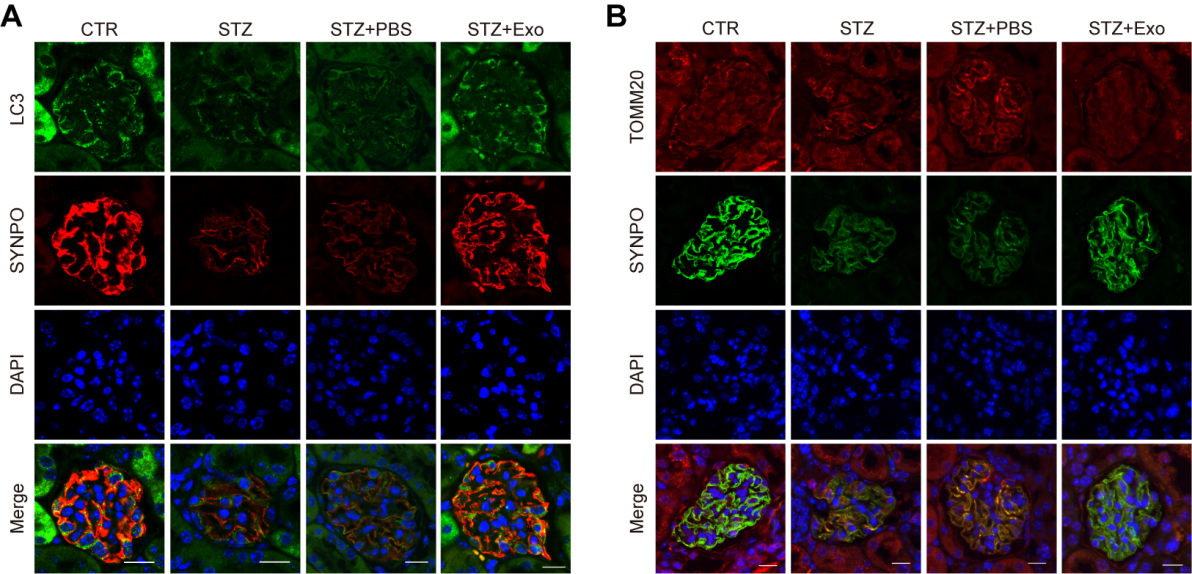


**Figure S2 Treg-Exos alleviate podocyte injury in diabetic mice.**

Immunofluorescence staining images of LC3 (A), TOMM20 (B) and SYNPO expression in the glomerular of diabetic mice injected with Treg-Exos. Scale bar, 20 μm. n=3 per group. Images are from one representative experiment from 3 independent experiments.


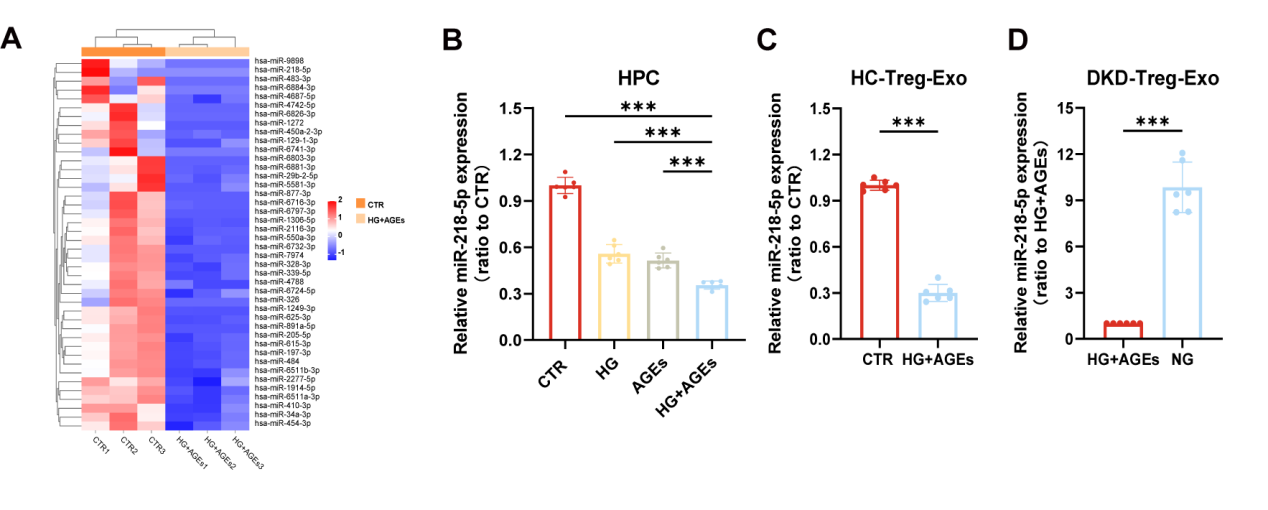


**Figure S3 Treg-Exos alleviate podocyte injury via miR-218-5p.**

A) A heatmap of downregulated miRNAs in podocytes treated with HG+AGEs. B) Quantitative PCR analysis of the expression of miR-218-5p in HPCs treated with HG or AGEs or HG+AGEs. n=6 per group. C) Quantitative PCR analysis of the expression of miR-218-5p in HC-Treg-Exos treated with HG+AGEs. n=6 per group. D) Quantitative PCR analysis of the expression of miR-218-5p in DKD-Treg-Exos treated with NG or HG+AGEs. n=6 per group. For all statistical plots, the data are presented as mean ± SD, the distinct dots are represented as the individual values of 6 replicates. *p* values were calculated by a two-tailed unpaired Student’s *t* test, one-way ANOVA and Tukey’s multiple comparison test. ****P < 0.001.*


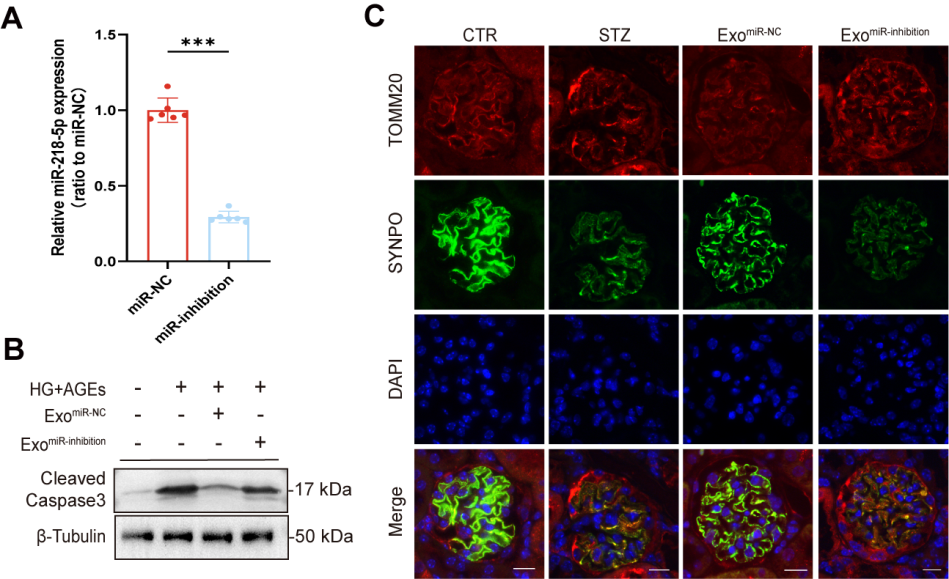


**Figure S4 Treg-Exos^miR-inhibition^ abolish the protective effect of Treg-Exos on podocytes**

A) Quantitative PCR analysis of the expression of miR-218-5p in Treg-Exos transfected with miRNA-NC or miR-218-5p-inhibition plasmids for 24 hours. n=6 per group. B) Western blot analysis of cleaved caspase3 expression in HPCs treated with Treg-Exos^miR-inhibition^ for 48 hours. n=3 per group. C) Immunofluorescence staining images of TOMM20 and SYNPO expression in the glomerular of diabetic mice injected with Treg-Exos^miR-inhibition^. Scale bar, 20 μm. n=3 per group. Images are from one representative experiment from 3 independent experiments. *p* values were calculated by a two-tailed unpaired Student’s *t* test. ****P < 0.001.*


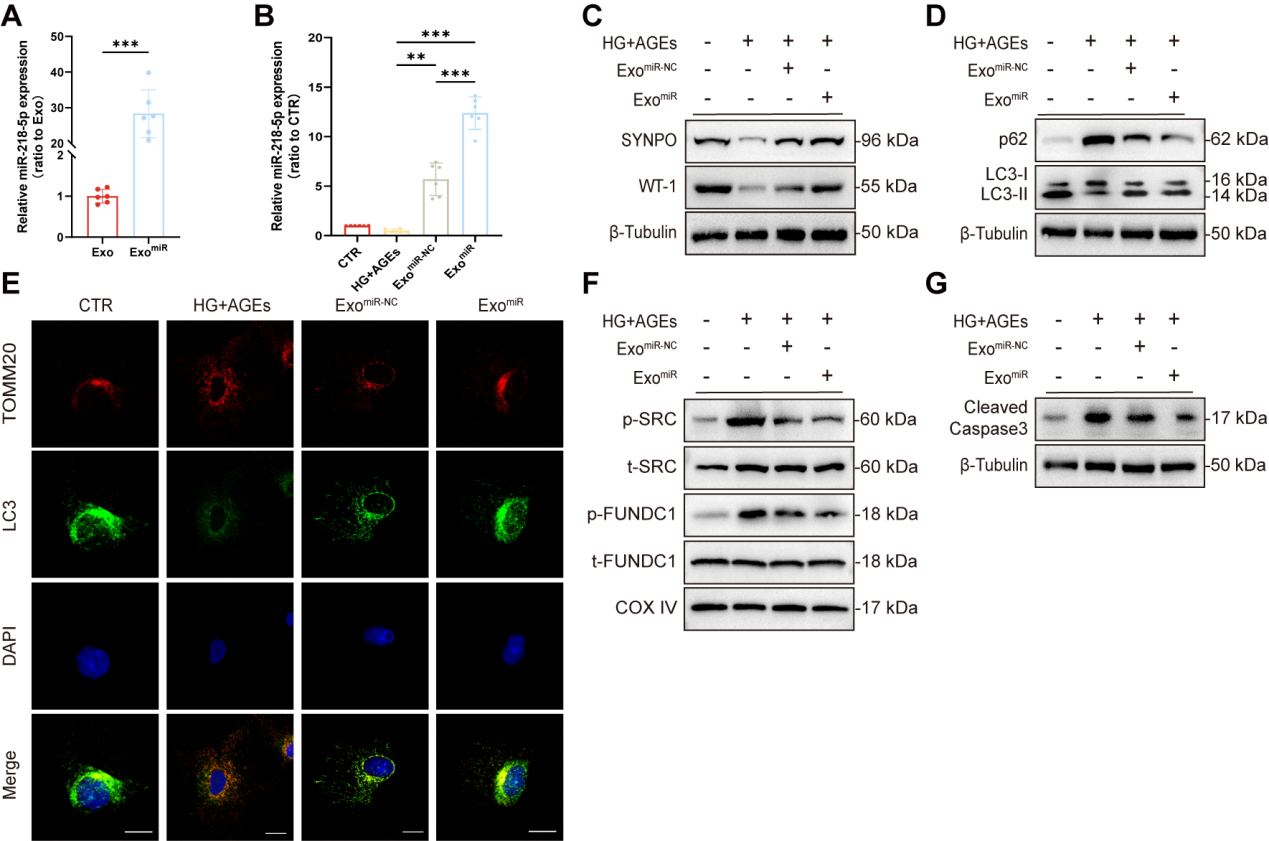


**Figure S5 Treg-Exos^miR^ alleviate podocyte injury induced by HG+AGEs.**

A) Quantitative PCR analysis of the expression of miR-218-5p in Treg-Exos transfected with miR-218-5p plasmids for 24 hours. n=6 per group. B) Quantitative PCR analysis of the expression of miR-218-5p in HPCs treated with Treg-Exos^miR^ for 24 hours. n=6 per group. Western blot analysis of SYNPO, WT-1 (C), p62 and LC3 conversion (D) expression in HPCs treated with Treg-Exos^miR^ for 48 hours. n=3 per group. E) Immunofluorescence staining images of TOMM20 and LC3 expression in HPCs treated with Treg-Exos^miR^ for 48 hours. Scale bar, 20 nm. n=3 per group. Western blot analysis of p-SRC, t-SRC, p-FUNDC1, t-FUNDC1 (F) and cleaved caspase3 (G) expression in HPCs treated with Treg-Exos^miR^ for 48 hours. n=3 per group. Images are from one representative experiment from 3 independent experiments. For all statistical plots, the data are presented as mean ± SD, the distinct dots are represented as the individual values of 6 replicates. *p* values were calculated by a two-tailed unpaired Student’s *t* test, one-way ANOVA and Tukey’s multiple comparison test. ***P < 0.01*, ****P < 0.001.*


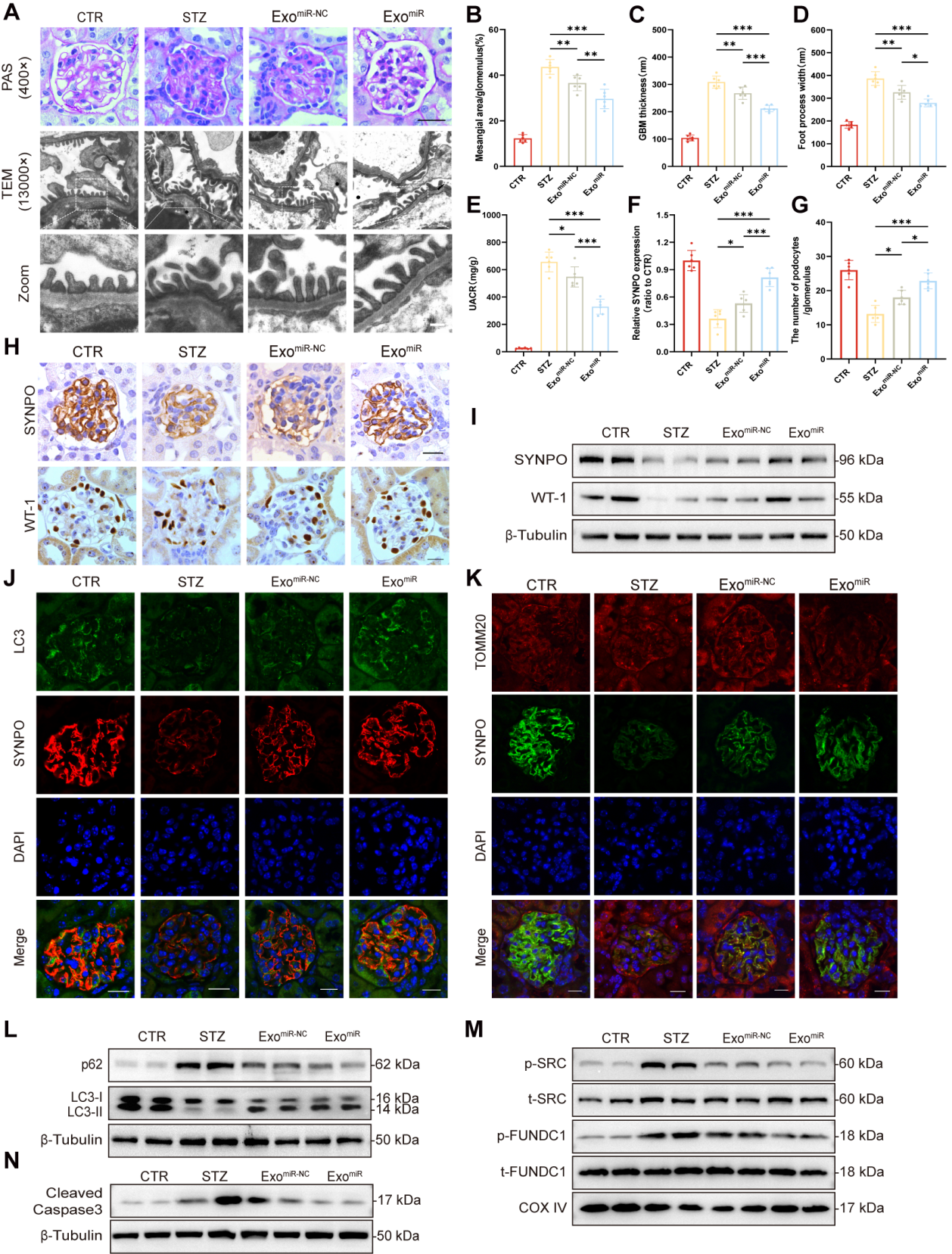


**Figure S6 Treg-Exos^miR^ alleviate podocyte injury in diabetic mice.**

A) Morphological examination of glomerular changes using PAS staining and TEM in diabetic mice injected with Treg-Exos^miR^. Scale bar, 20 μm (PAS), 250 nm (TEM), 25 nm (Zoom). n=6 per group. B) Quantification of the mesangial area in diabetic mice injected with Treg-Exos^miR^. n=6 per group. C) Quantification of GBM thickness in diabetic mice injected with Treg-Exos^miR^. n=6 per group. D) Mean width of the podocyte foot processes in diabetic mice injected with Treg-Exos^miR^. n=6 per group. E) Urinary albumin-to-creatinine ratios in diabetic mice injected with Treg-Exos^miR^. n=6 per group. H) IHC analysis of SYNPO and WT-1 expression in the glomerular of diabetic mice injected with Treg-Exos^miR^. Scale bar, 20 μm. n=6 per group. F) Quantitative analysis of the density of SYNPO in (H). n=6 per group. G) Quantitative analysis of the number of podocytes per glomerulus in (H). n=6 per group. I) Western blot analysis of SYNPO and WT-1 expression in renal cortex of diabetic mice injected with Treg-Exos^miR^. n=3 per group. Immunofluorescence staining images of LC3 (J), TOMM20 (K) and SYNPO expression in the glomerular of diabetic mice injected with Treg-Exo^miR^. Scale bar, 20 μm. n=3 per group. Western blot analysis of p62, LC3 conversion (L), p-SRC, t-SRC, p-FUNDC1, t-FUNDC1 (M) and cleaved caspase3 (N) expression in renal cortex of diabetic mice injected with Treg-Exos^miR^. n=3 per group. Images are from one representative experiment from 3 to 6 independent experiments. For all statistical plots, the data are presented as mean ± SD, the distinct dots are represented as the individual values of 6 replicates. *p* values were calculated by one-way ANOVA and Tukey’s multiple comparison test. **P < 0.05, **P < 0.01*, ****P < 0.001.*


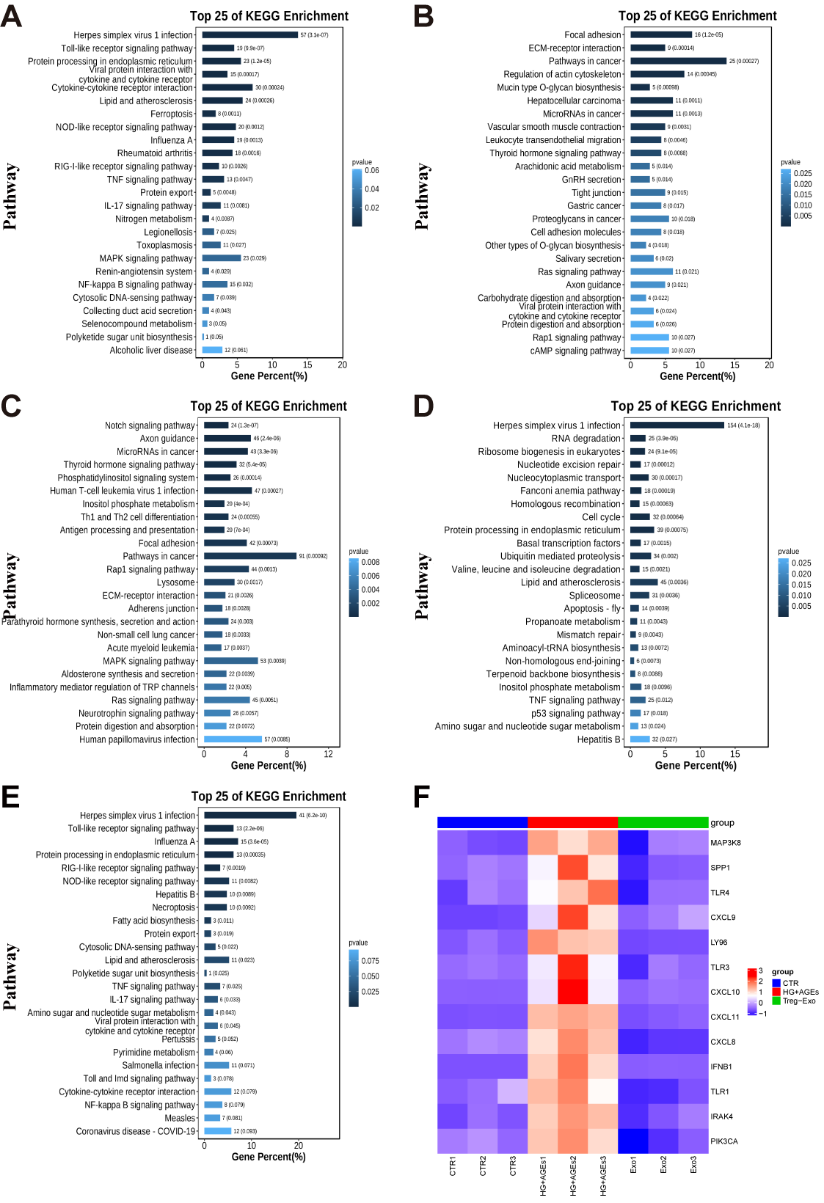


**Figure S7 Bioinformatics analysis of podocytes treated with Treg-Exos^miR^.**

A) KEGG pathway analysis of the upregulated DEGs in the HG+AGEs *vs.* CTR groups. B) KEGG pathway analysis of the downregulated DEGs in the HG+AGEs *vs.* CTR groups. C) KEGG pathway analysis of the upregulated DEGs in the Treg-Exos^miR^ *vs.* HG+AGEs groups. D) KEGG pathway analysis of the downregulated DEGs in the Treg-Exos^miR^ *vs.* HG+AGEs groups. E) KEGG pathway analysis of the intersection DEGs of (A) and (D). F) A heatmap of Toll like receptor signaling pathway genes in the Treg-Exos^miR^ groups.


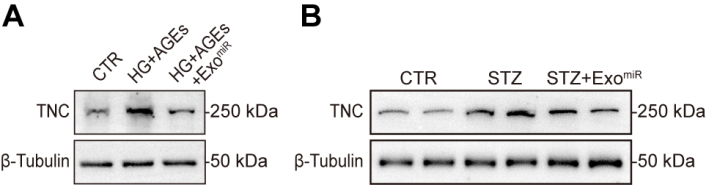


**Figure S8 Treg-Exos^miR^ decline the expression of TNC.**

A)Western blot analysis of TNC expression in HPCs treated with Treg-Exos^miR^ for 48 hours. n=3 per group. B) Western blot analysis of TNC expression in diabetic mice injected with Treg-Exos^miR^. n=3 per group. Images are from one representative experiment from 3 independent experiments.


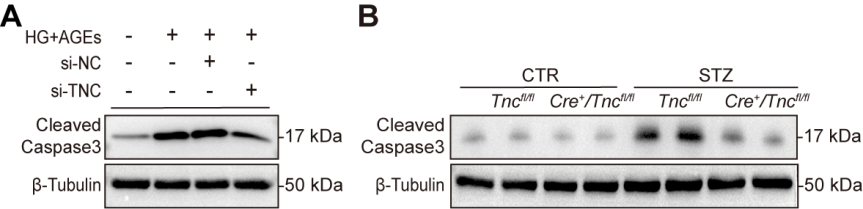


**Figure S9 Knocking down TNC alleviate podocyte injury both in vitro and in vivo.**

A) Western blot analysis of cleaved caspase3 in HPCs transfected with si-TNC for 48 hours. n=3 per group. B) Western blot analysis of cleaved caspase3 in renal cortex of *Nphs2-Cre^+^/Tnc^fl/fl^* diabetic mice. n=3 per group. Images are from one representative experiment from 3 independent experiments.


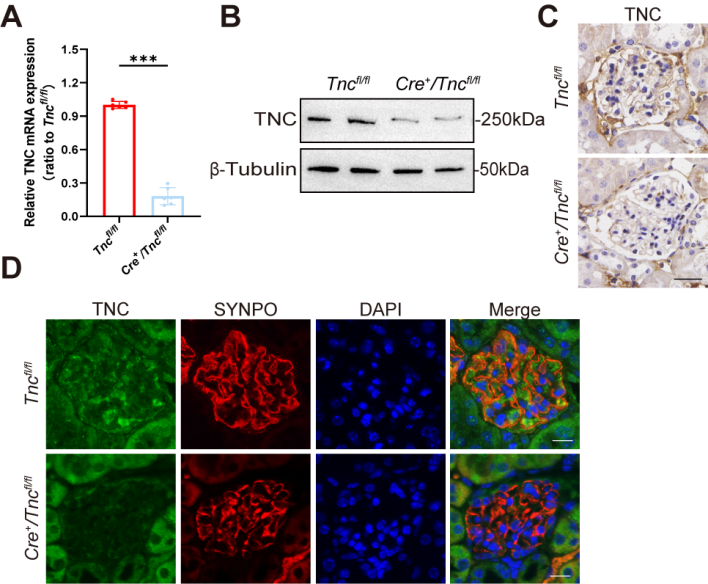


**Figure S10** **Validation of *Nphs2-Cre^+^/Tnc^fl/fl^* mice.**

A) Quantitative PCR analysis of the expression of TNC mRNA in renal cortex of *Nphs2-Cre^+^/Tnc^fl/fl^* mice. n=6 per group. B) Western blot analysis of TNC expression in renal cortex of *Nphs2-Cre^+^/Tnc^fl/fl^* mice. n=3 per group. C) IHC analysis of TNC expression in the glomerular of *Nphs2-Cre^+^/Tnc^fl/fl^* mice. Scale bar, 20 μm. n=3 per group. D) Fluorescence staining of TNC and SYNPO expression in the glomerular of *Tnc^fl/fl^* and *Nphs2-Cre^+^/Tnc^fl/fl^* mice. Scale bar, 20 μm. n=3 per group. Images are from one representative experiment from 3 independent experiments. For all statistical plots, the data are presented as mean ± SD, the distinct dots are represented as the individual values of 6 replicates. *p* values were calculated by a two-tailed unpaired Student’s *t* test. ****P < 0.001.*


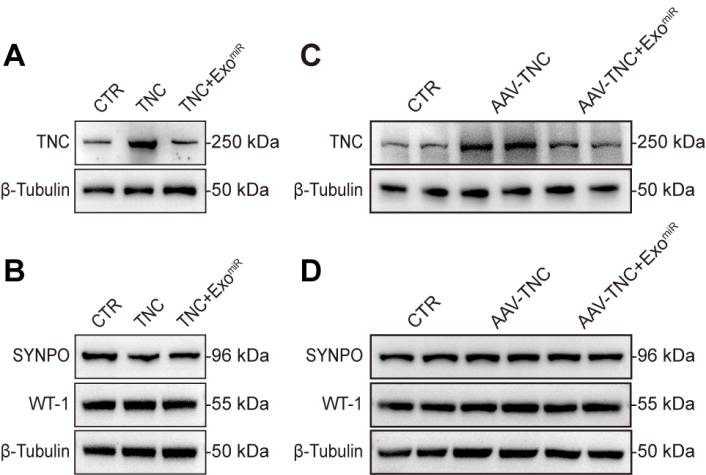


**Figure S11 Overexpression of TNC in vitro and vivo.**

Western blot analysis of TNC (A), SYNPO and WT-1 (B) expression in HPCs transfected with TNC plasmids and treated with Treg-Exos^miR^ for 48 hours. n=3 per group. Western blot analysis of TNC (C), SYNPO and WT-1 (D) expression in renal cortex of mice injected with AAV-TNC and Treg-Exos^miR^. n=3 per group. Images are from one representative experiment from 3 independent experiments.


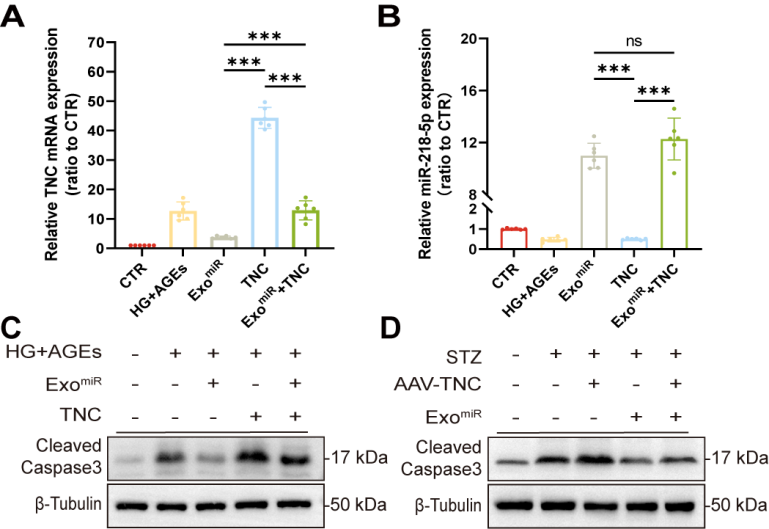


**Figure S12 Treg-Exos^miR^ reverse podocyte injury exacerbated by TNC overexpression in vitro.**

Quantitative PCR analysis of the expression of TNC mRNA (A) and miR-218-5p (B) in HPCs treated with Treg-Exos^miR^ or/and transfected with TNC plasmids for 24 hours. n=6 per group. C) Western blot analysis of cleaved caspase3 expression in HPCs treated with Treg-Exos^miR^ or/and transfected with TNC plasmids for 48 hours. n=3 per group. D) Western blot analysis of cleaved caspase3 expression in renal cortex of diabetic mice injected with Treg-Exos^miR^ or/and AAV-TNC. Images are from one representative experiment from 3 independent experiments. For all statistical plots, the data are presented as mean ± SD, the distinct dots are represented as the individual values of 6 replicates. *p* values were calculated by one-way ANOVA and Tukey’s multiple comparison test. ns *P* *>0.05,* ****P < 0.001.*


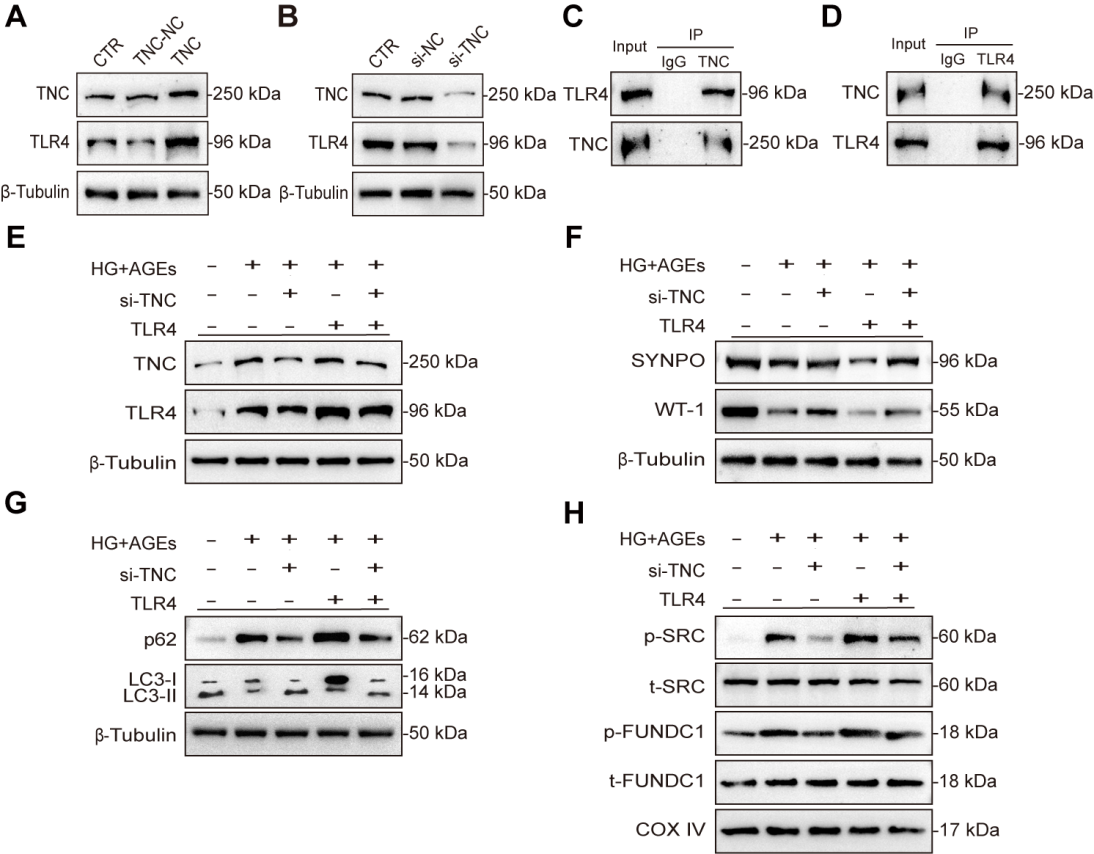


**Figure S13 TLR4 overexpression reverses the effect of knocking down TNC in podocytes.**

A, B) Western blot analysis of TNC and TLR4 expression in HG+AGEs-treated HPCs transfected with TNC plasmids or si-TNC for 48 hours. n=3 per group. (C, D) TNC and TLR4 interaction in HG+AGEs-treated HPCs was assessed by Co-immunoprecipitation experiments. n=3 per group. Western blot analysis of TNC, TLR4 (E), SYNPO, WT-1 (F), p62 and LC3 conversion (G), p-SRC, t-SRC, p-FUNDC1 and t-FUNDC1 (H) in HPCs transfected with TLR4 plasmids or si-TNC for 48 hours. n=3 per group. Images are from one representative experiment from 3 independent experiments.


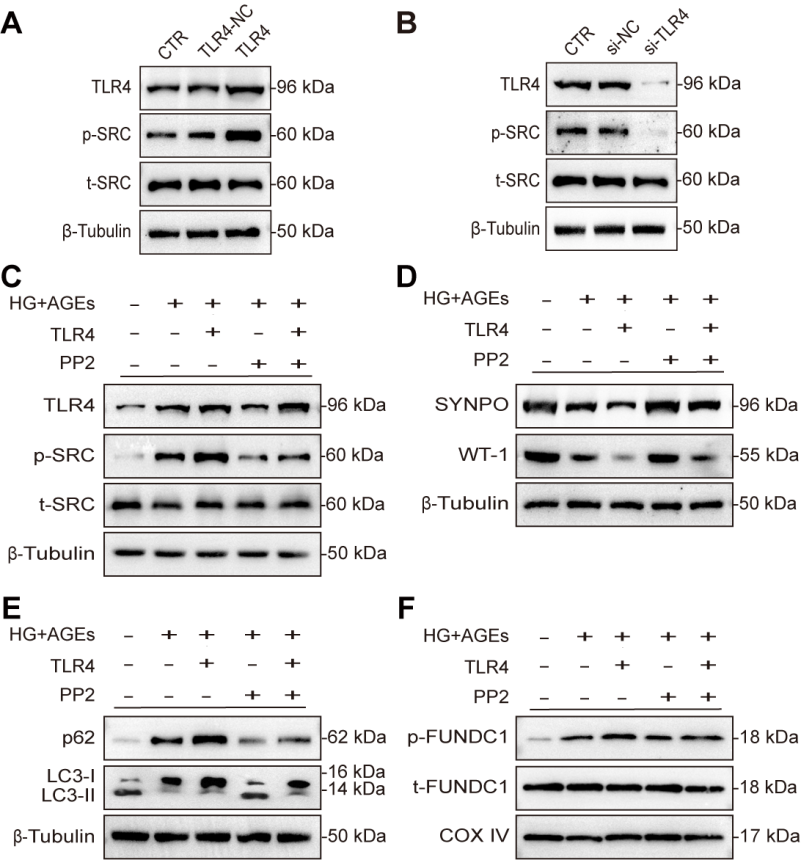


**Figure S14 PP2 reverses podocyte injury induced by TLR4 overexpression in vitro.**

A, B) Western blot analysis of TLR4, p-SRC and t-SRC expression in HG+AGEs-treated HPCs transfected with TLR4 plasmids or si-TLR4 for 48 hours. n=3 per group. Western blot analysis of TLR4, p-SRC, t-SRC (C), SYNPO, WT-1 (D), p62 and LC3 conversion (E), p-FUNDC1 and t-FUNDC1 (F) in HPCs treated with TLR4 plasmids or PP2 for 48 hours. n=3 per group. Images are from one representative experiment from 3 independent experiments.


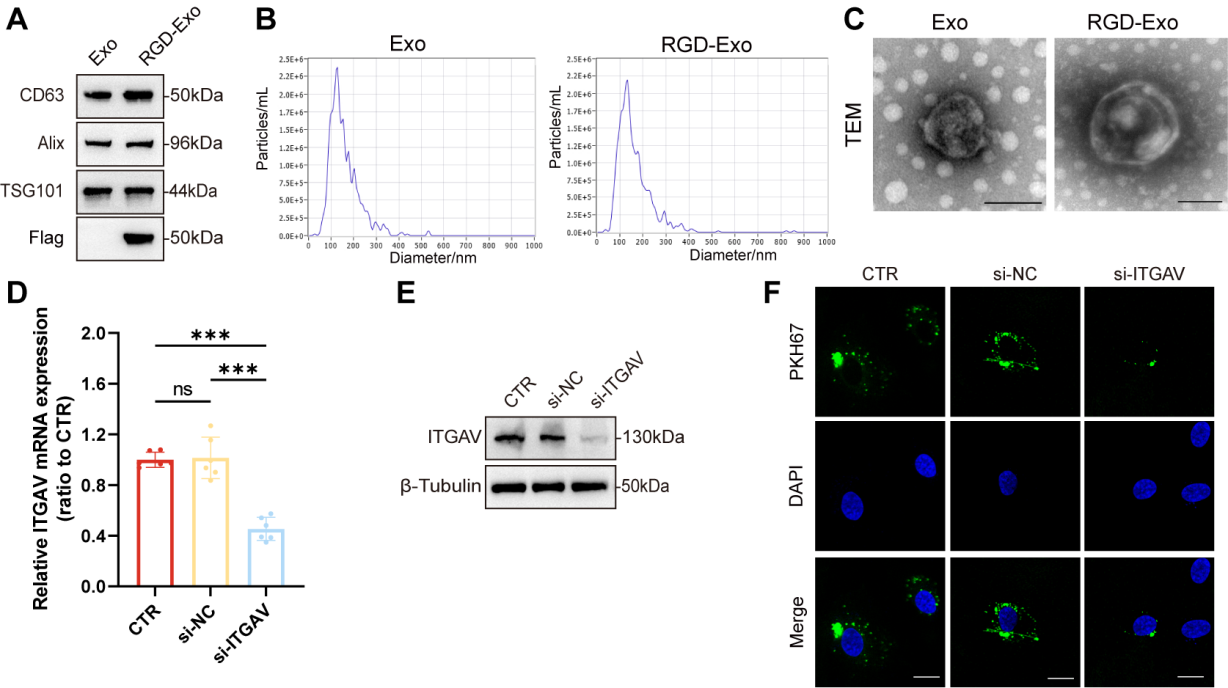


**Figure S15 Analysis of RGD-Treg-Exos in vitro.**

A) Western blot analysis of Treg-Exos biomarkers (CD63, Alix and TSG101) and Flag expression in Treg-Exos and RGD-Treg-Exos. n=3 per group. Identification of Treg-Exos and RGD-Treg-Exos by NTA (**B**) and TEM (**C**). Scale bar, 100 nm. n=3 per group. D) Quantitative PCR analysis of the expression of ITGAV mRNA in HPCs transfected with si-ITGAV for 24 hours. n=6 per group. E) Western blot analysis of ITGAV in HPCs transfected with si-ITGAV for 48 hours. n=3 per group. F) Fluorescence staining images of PKH67-labelled RGD-Treg-Exos ingested by HPCs after 24h incubation. Scale bar, 20 nm. n=3 per group. Images are from one representative experiment from 3 independent experiments. For all statistical plots, the data are presented as mean ± SD, the distinct dots are represented as the individual values of 6 replicates. *p* values were calculated by one-way ANOVA and Tukey’s multiple comparison test. ns *P > 0.05*, ****P < 0.001.*


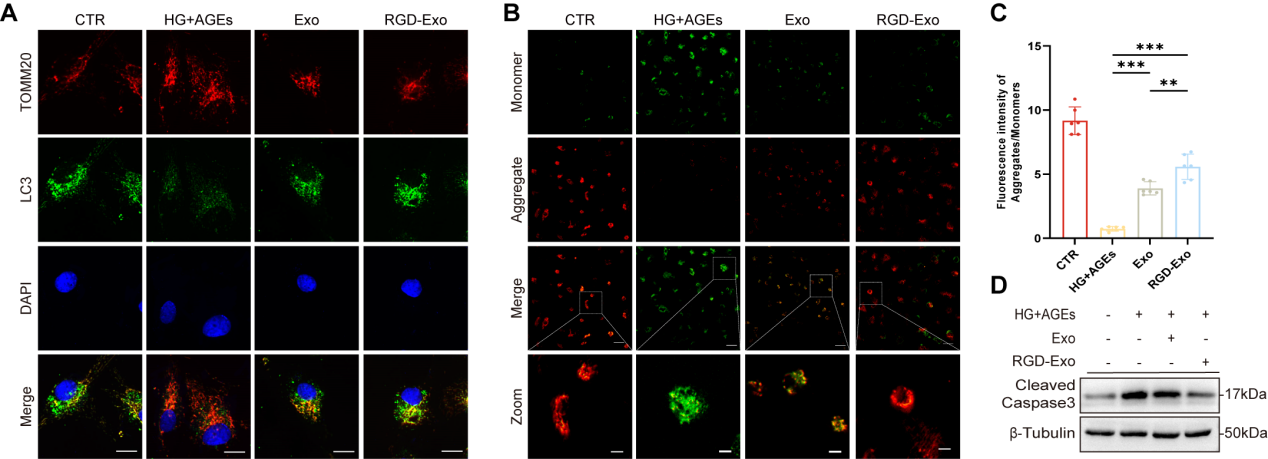


**Figure S16 RGD-Treg-Exos alleviate podocyte injury better in vitro.**

A) Immunofluorescence staining images of TOMM20 and LC3 expression in HPCs treated with Treg-Exos or RGD-Treg-Exos for 48 hours. Scale bar,20 nm. n=3 per group. B) Fluorescence staining images of mitochondria membrane potential by JC-1 staining in HPCs treated with Treg-Exos or RGD-Treg-Exos for 48 hours. Scale bar, 20 nm (Merge), 2 nm (Zoom). n=6 per group. C) Quantitative statistics of fluorescence intensity of Aggregate/Monomer in (B) results. n=6 per group. E) Western blot analysis of cleaved caspase3 expression in HPCs treated with Treg-Exos or RGD-Treg-Exos for 48 hours. n=3 per group. Images are from one representative experiment from 3 to 6 independent experiments. For all statistical plots, the data are presented as mean ± SD, the distinct dots are represented as the individual values of 6 replicates. *p* values were calculated by one-way ANOVA and Tukey’s multiple comparison test. ***P < 0.01*, ****P < 0.001.*


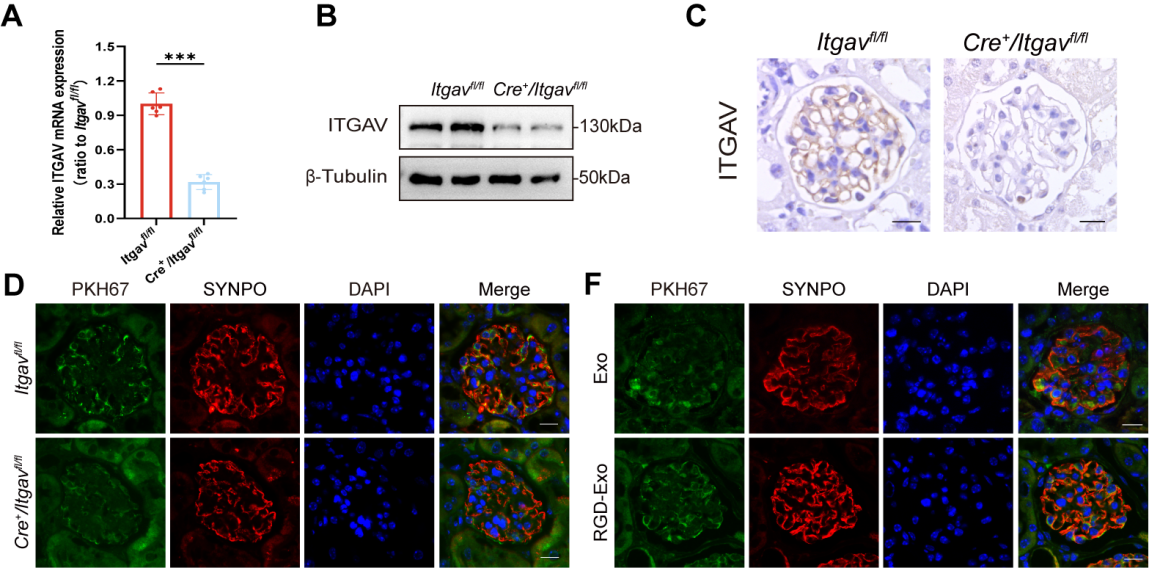


**Figure S17 Targeted exploration of RGD-Treg-Exos in mice in vivo.**

A) Quantitative PCR analysis of the expression of ITGAV mRNA in renal cortex of *Nphs2-Cre^+^/Itgav^fl/fl^* mice. n=6 per group. B) Western blot analysis of ITGAV expression in renal cortex of *Nphs2-Cre^+^/Itgav^fl/fl^* mice. n=3 per group. C) IHC analysis of ITGAV expression in the glomerular of *Nphs2-Cre^+^/Itgav^fl/fl^* mice. n=3 per group. D) Colocalization fluorescence staining images of PKH67-labeled RGD-Treg-Exos (green) and a podocyte marker SYNPO (red) in the glomerular of *Nphs2-Cre^+^/Itgav^fl/fl^* mice. Scale bar, 20 μm. n=3 per group. F) Colocalization fluorescence staining images of PKH67-labeled Treg-Exos or RGD-Treg-Exos (green) and a podocyte marker SYNPO (red) in the glomerular of diabetic mice. Scale bar, 20 μm. n=3 per group. Images are from one representative experiment from 3 independent experiments. For all statistical plots, the data are presented as mean ± SD, the distinct dots are represented as the individual values of 6 replicates. *p* values were calculated by a two-tailed unpaired Student’s *t* test. ****P < 0.001.*

**
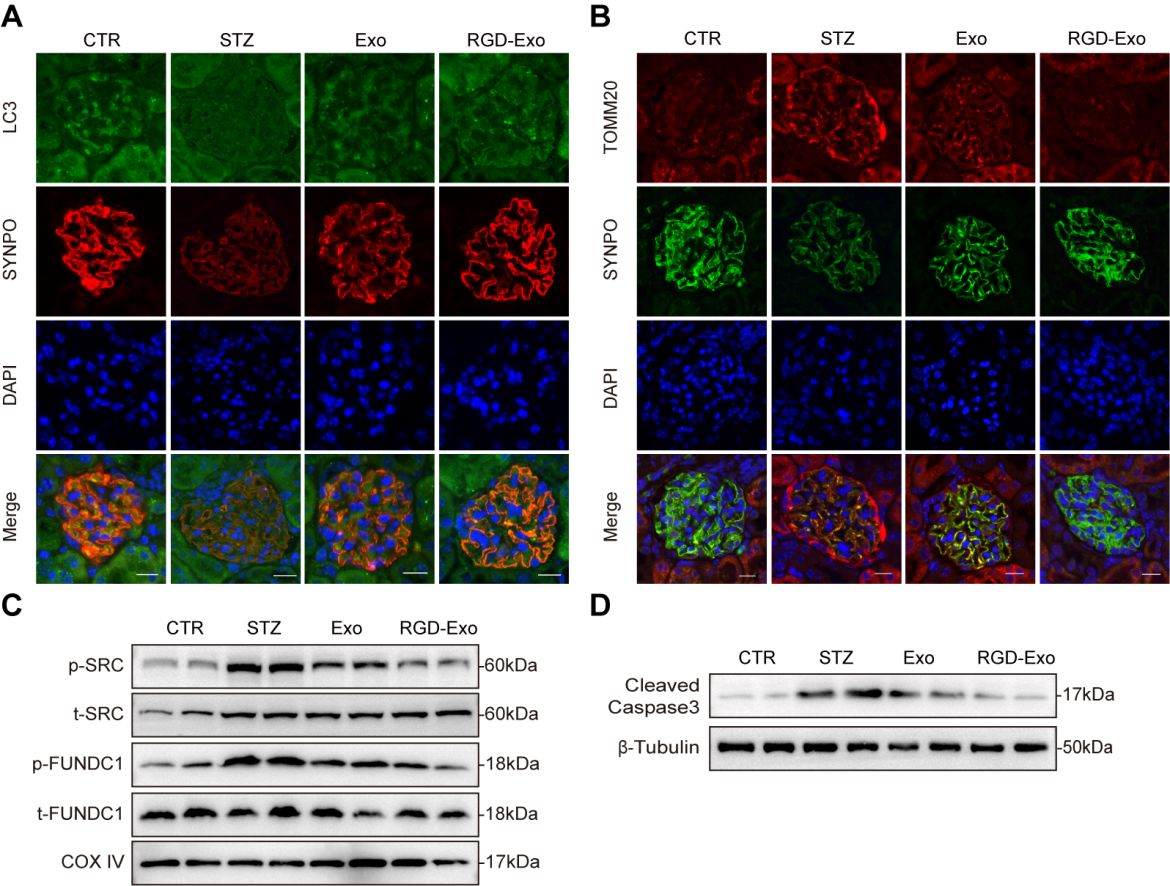
**

**Figure S18 RGD-Treg-Exos are more effective in attenuating podocyte injury in vivo.**

Immunofluorescence staining images of LC3 (A), TOMM20 (B) and SYNPO expression in the glomerular of diabetic mice injected with Treg-Exos or RGD-Treg-Exos. Scale bar, 20 μm. n=3 per group. Western blot analysis of p-SRC, t-SRC, p-FUNDC1, t-FUNDC1(C) and cleaved caspase3 (D) expression in renal cortex of diabetic mice injected with Treg-Exos or RGD-Treg-Exos. n=3 per group. Scale bar, 20 μm. n=3 per group. Images are from one representative experiment from 3 independent experiments.


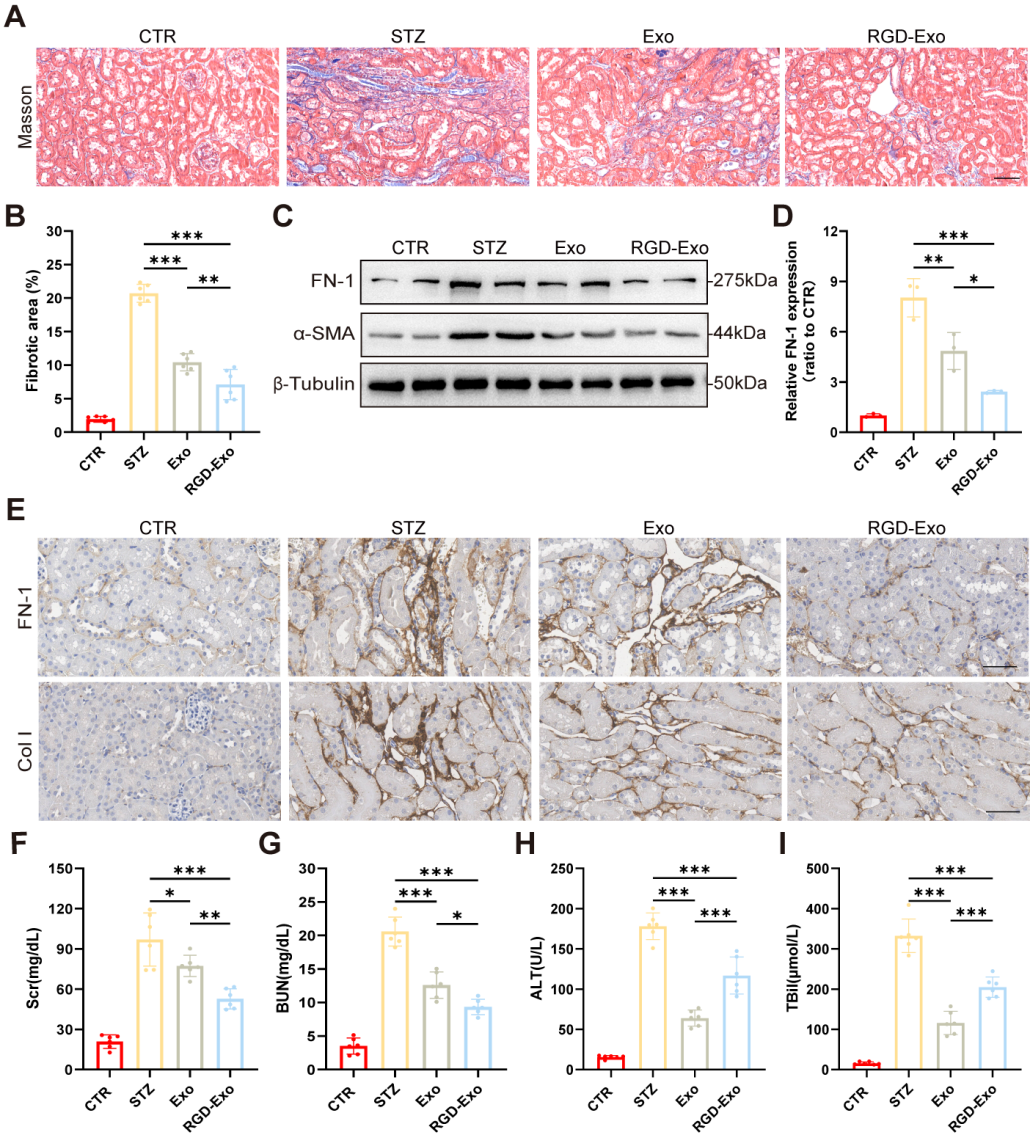


**Figure S19** **RGD-Treg-Exos alleviate renal fibrotic response in vivo.**

(A) Masson staining of the kidney of diabetic mice injected with Exos or RGD-Exos. Scale bar, 50 μm. n=6 per group. (B) Quantification of fibrotic area in (A). n=6 per group. (C) Western blot analysis of FN-1 and α-SMA expression in the kidney of diabetic mice injected with Exos or RGD-Exos. n=6 per group. (D) Quantification of FN-1 expression in (C). n=6 per group. (E) IHC analysis of FN-1 and Col I expression in the kidney of diabetic mice injected with Exos or RGD-Exos. Scale bar, 50 μm. n=3 per group. The serum levels of Scr (F), BUN (G), ALT (H), and TBil (I) were measured. n=6 per group. Images are from one representative experiment from 3 to 6 independent experiments. For all statistical plots, the data are presented as mean ± SD, the distinct dots are represented as the individual values of 6 replicates. *p* values were calculated by one-way ANOVA and Tukey’s multiple comparison test. **P < 0.05*, ***P < 0.01*, ****P < 0.001*.

**Supplementary Table 1. Characteristics of individuals enrolled in the study.**

Data were presented as group mean ± SEM. M, male. F, female.

|  | Healthy control（n=6） | RCC patients  （n=6） | DKD patients  （n=6） |
| --- | --- | --- | --- |
| Sex | 3M/3F | 4M/2F | 3M/3F |
| Age (yr) | 49.00±3.01 | 62.50±2.20 | 60.67±2.70 |
| UACR（mg/g） | 7.91±1.88 | 12.53±2.54 | 379.30±39.79 |
| Serum creatinine（μmol/L） | 70.80±3.82 | 66.68±2.75 | 173.20±12.93 |

**Supplementary Table 2. Human primers used for RT-qPCR**

| Gene | Sequence |
| --- | --- |
| miR-218-5p | F：GCGCGTTGTGCTTGATCTAA |
|  | R：AGTGCAGGGTCCGAGGTATT |
| miR-29b-2-5p | F：GCGCTGGTTTCACATGGTG |
|  | R：AGTGCAGGGTCCGAGGTATT |
| TNC | F：TCCCAGTGTTCGGTGGATCT |
|  | R：TTGATGCGATGTGTGAAGACA |
| ELFN2 | F：TCAAAGCCGTGCTCTACTCCT |
|  | R：CGTCAGGTTGCTGAGCTTG |
| TFAP2E | F：GCCTCCTAGACCAGTCCGT |
|  | R：CTCACCAGGATTTGTGATGCC |
| COL1A1 | F：GAGGGCCAAGACGAAGACATC |
|  | R：CAGATCACGTCATCGCACAAC |
| CACNA1G | F：TGTCTCCGCACGGTCTGTAA |
|  | R：AAGCCGGTTCCAAGTGTCTC |
| ISLR2 | F：GCGGACTGCGCTTACAAAG |
|  | R：GTGGCTCAGATCGAGGTTCTT |
| KCNIP3 | F：CAGTCTCTCTACAGGGGCTTT |
|  | R：AGAACTGCGCGTAAATGAGTTT |
| GAPDH | F：GGAGCGAGATCCCTCCAAAAT |
|  | R：GGCTGTTGTCATACTTCTCATGG |
| U6 | F：CTCGCTTCGGCAGCACA |
|  | R：AACGCTTCACGAATTTGCGT |
| Stem-loop | GTCGTATCCAGTGCAGGGTCCGAGGTATTCGCACTGGATACGAC |
| ITGAV | F：GCTGTCGGAGATTTCAATGGT |
|  | R：TCTGCTCGCCAGTAAAATTGT |

**Supplementary Table 3. Mouse primers used for RT-qPCR**

| Gene | Sequence |
| --- | --- |
| miR-218-5p | F：GCGCGTTGTGCTTGATCTAA |
|  | R：AGTGCAGGGTCCGAGGTATT |
| miR-29b-2-5p | F：GCTGGTTTCACATGGTGGCT |
|  | R：AGTGCAGGGTCCGAGGTATT |
| Stem-loop | GTCGTATCCAGTGCAGGGTCCGAGGTATTCGCACTGGATACGAC |
| U6 | F：CTCGCTTCGGCAGCACA |
|  | R：AACGCTTCACGAATTTGCGT |
| ITGAV | F：CGGGTCCCGAGGGAAGTTA |
|  | R：TGGATGAGCATTCACATTTGAGA |
| GAPDH | F：AGGTCGGTGTGAACGGATTTG |
|  | R：GGGGTCGTTGATGGCAACA |

**Supplementary Table 4. Flow cytometry antibody information.**

| Antibodies | Manufacturer | Fluorescence | Dilution |
| --- | --- | --- | --- |
| CD4 | eBiosciences（Thermo Fisher） | FITC | 5μl/Test |
| CD25 | eBiosciences | PE | 5μl/Test |
| CD127 | eBiosciences | APC | 5μl/Test |

**Supplementary Table 5. Antibody information.**

| Antibodies | Manufacturer | Source | Dilution |
| --- | --- | --- | --- |
| COX IV | Abcam（Cambridge，UK） | Mouse | WB：1：5000 |
| WT1 | Abcam | Rabbit | WB：1：2000  IHC: 1：200 |
| ITGAV | Abcam | Rabbit | WB：1：2000  IHC：1：200 |
| PINK | Abcam | Rabbit | WB：1：2000 |
| Parkin | Abcam | Rabbit | WB：1：2000 |
| BNIP3 | Abcam | Rabbit | WB：1：2000 |
| Nix | Abcam | Rabbit | WB：1：2000 |
| CD63 | Abcam | Rabbit | WB：1：1000 |
| Alix | Abcam | Rabbit | WB：1：1000 |
| TSG101 | Abcam | Rabbit | WB：1：1000 |
| Calnexin | Abcam | Rabbit | WB：1：4000 |
| SRC | ABclonal（Wuhan，China） | Rabbit | WB：1：2000 |
| p-SRC | ABclonal | Rabbit | WB：1：2000 |
| cleaved-caspase3 | ABclonal | Rabbit | WB：1：2000 |
| Caspase3 | ABclonal | Rabbit | WB：1：2000 |
| SQSTM1/p62 | ABclonal | Rabbit | WB：1：2000 |
| TNC | ABclonal | Rabbit | WB：1：2000  IP：2 µg per 500 µg of total protein |
| p-FUNDC1 | Huabio（Hangzhou, China） | Rabbit | WB：1：2000 |
| FN1 | Proteintech（Wuhan，China） | Rabbit | WB：1：10000 |
| α-SMA | Proteintech | Rabbit | WB：1：10000 |
| TGF-β | Proteintech | Rabbit | WB：1：5000 |
| β-Tubulin | Proteintech | Rabbit | WB：1：50000 |
| TLR4 | Proteintech | Rabbit | WB：1：4000  IP：2 µg per 500 µg of total protein |
| DYKDDDDK tag | Proteintech | Mouse | WB：1：10000 |
| CoraLite®594-conjugated TOM20 | Proteintech | Rabbit | IF：1：200 |
| SYNPO | Santa Cruz（Texas, California，USA） | Mouse | WB：1：100  IHC：1：50  IF：1：50 |
| LC3A/B (D3U4C) XP® Rabbit mAb | Cell Signaling Technology (CST) (Danvers, MA, United States) |  | WB：1：1000  IF：1：200 |
| HRP Goat Anti-Rabbit IgG （H+L） | Boster（Wuhan，China） | Goat | WB：1：5000 |
| HRP Goat Anti-Mouse IgG （H+L） | Boster | Goat | WB：1：5000 |
| CoraLite594-conjugated Goat Anti-Mouse IgG（H+L） | Proteintech | Goat | IF：1：200 |
| CoraLite488-conjugated Goat Anti- Mouse IgG（H+L) | Proteintech | Goat | IF：1：200 |
| CoraLite488-conjugated Goat Anti-Rabbit IgG（H+L） | Proteintech | Goat | IF：1：200 |
